# Integrated pulse scope for tunable generation and intrinsic characterization of structured femtosecond laser

**DOI:** 10.1038/s41598-021-87938-w

**Published:** 2021-05-06

**Authors:** Tiancheng Huo, Li Qi, Jason J. Chen, Yusi Miao, Zhongping Chen

**Affiliations:** 1 Beckman Laser Institute, University of California, Irvine, Irvine, CA 92617 USA; 2 The Edwards Lifesciences Center for Advanced Cardiovascular Technology, Department of Biomedical Engineering, University of California, Irvine, Irvine, CA 92617 USA

**Keywords:** Optics and photonics, Fibre lasers, Ultrafast lasers

## Abstract

Numerous techniques have been demonstrated for effective generation of orbital angular momentum-carrying radiation, but intracavity generation of continuously tunable pulses in the femtosecond regime remains challenging. Even if such a creation was realized, the generated pulses—like all pulses in reality—are complex and transitory objects that can only be comprehensively characterized via multidimensional spaces. An integrated lasing system that generates pulses while simultaneously quantifies them can achieve adaptive pulse tailoring. Here, we report a femtosecond pulse scope that unifies vector vortex mode-locked lasing and vectorial quantification. With intracavity-controlled Pancharatnam-Berry phase modulation, continuous and ergodic generation of spirally polarized states along a broadband higher-order Poincaré sphere was realized. By intrinsically coupling a two-dimensional polarization-sensitive time-scanning interferometer to the laser, multidimensional spatiotemporal features of the pulse were further visualized. The proposed methodology paves the way for design optimization of ultrafast optics by integrating complex femtosecond pulse generation and structural customization, facilitating its applications in optical physics research and laser-based manufacturing.

## Introduction

A photon carries an orbital angular momentum (OAM) and a spin angular momentum (SAM), which are not strictly distinguishable and separately conserved^1^. Under the generally accepted paraxial approximation, the analogy between the time dependent Schrodinger equation and optical scalar wave equation^2^ suggests that the linearly polarized Laguerre-Gaussian mode with null radial index will carry a well-defined OAM, which may be associated with the phase factor for an optical vortex along its axis^3^. OAM-carrying beams have recently attracted major attention in the theoretical research^4,5^ and for its applications in optomechanics^6^, optical communications^7^, particle acceleration^8^, microscopy^9,10^, optical trapping and tweezing^11,12^, material processing^13^, remote sensing^14,15^, and quantum sciences^16,17^.

A range of external-cavity techniques have been developed for creating OAM-carrying beams by using spatial light modulators^18,19^, Q-plates^20,21^ or optical metasurfaces^22^, sectored spatially varying retarder^23^, and diffractive optics^24,25^. Due to insertion loss, polarization dependency, and dispersion, however, applying these techniques to a broadband source has been problematic. There even exists a paradox that modulating any freedom of a laser (e.g., polarization, spectrum, wavefront) would inevitably affect one or the other. Since the recent introduction of the helical modes of light^26–29^, researchers have been focusing on creating OAM-carrying pulses via an intracavity means as all spatial and spectral freedoms of the pulse intrinsically interact with each other. In an intracavity-controlled laser, mode-locking is typically employed to generate ultrashort vector vortex pulses in the picosecond or femtosecond range^30–35^. Although a variety of cavity designs have been proposed, a tunable OAM-carrying femtosecond laser with continuous and ergodic Pancharatnam-Berry phase modulation within the cavity would further allow for the generation of extremely short, refined, and high energy structured pulses.

While pulse characterization is as essential as pulse generation in ultrafast optics, comprehensive spatiotemporal quantification of a femtosecond pulse remains challenging because of its short duration and broadband nature. This is even more difficult if the pulse has a complex vectorial structure. Even though state-of-the-art streak cameras have reached a temporal resolution of ~ 100 fs^36^, direct pulse measurement is still limited by the photodetector response time. Among the indirect measurement approaches, the dechirped pulse duration is commonly tested through intensity correlation^37^. Spectral analysis methods, including frequency-resolved optical gating (FROG)^38^, spectral-phase interferometry for direct electric-field reconstruction (SPIDER)^39^, multiphoton intrapulse interference phase scan (MIIPS)^40^_,_ and dispersion scan^41^, can resolve the temporal profile and the relative spectral phases, but the spatial and polarization profile cannot be fully acquired even with recent modifications^42–48^. Nevertheless, characterization of wideband vector vortex beams has only been partially and qualitatively realized^49,50^, in which the spectral phase and temporal information are lost.

In this study, we report an integrated system for intracavity generation and multidimensional characterization of broadband OAM-carrying femtosecond pulses coupled with a cylindrically symmetric polarization structure that can be mapped onto the higher-order Poincaré (HOP) sphere state (HOP_SS_). We first demonstrate a novel V-shaped design for modulating Pancharatnam-Berry phase within the cavity that allows for passive mode-locking, high switching efficiency, ultra clean, and broadband output. To comprehensively evaluate the pulse quality, we then present a polarization-sensitive spatiotemporal pulse scope for characterizing the generated beams. The proposed system will provide complete knowledge of complex pulses and be used for real-time adaptive optimization and generation of mode-locked laser.

## Results

### Intracavity-controlled OAM-carrying femtosecond laser

**Figure 1:**
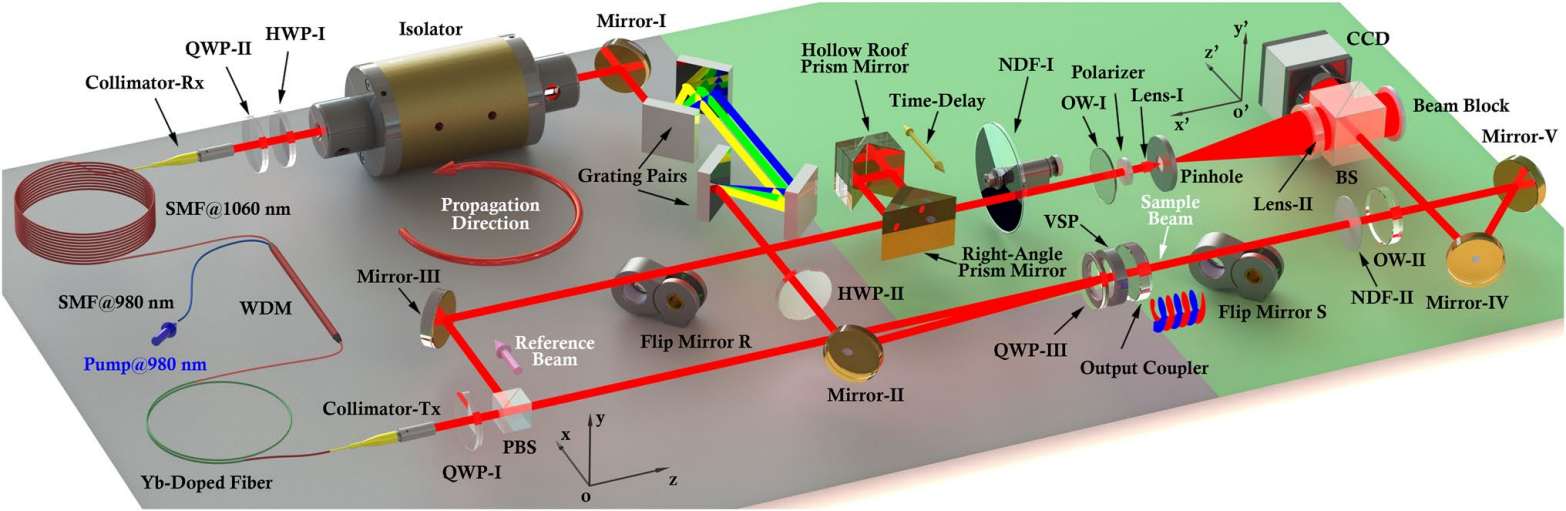
Schematic diagram of the integrated pulse scope. The gray portion of the background floor highlights the intracavity-controlled femtosecond laser, whereas the green portion of that describes the polarization-sensitive spatiotemporal characterization system. VSP = variable spiral plate; QWP = quarter wave plate; HWP = half wave plate; SMF = single mode fiber; WDM = wavelength division multiplexer; BS = beamsplitter; PBS = polarizing beamsplitter; OW = optical window; NDF = neutral density filter. The red circular arrow denotes the direction of the cavity modes.

Figure 1 depicts the schematic diagram of the pulse scope integrated with intracavity-controlled OAM-carrying laser and the characterization system, in which two reference systems, the global associated with the laboratory and the local with the charge-coupled device (CCD), are denoted by *oxyz* and *o’x’y’z’*, respectively. The positive direction of *oz* and *o’z’* refers to the propagation direction of the sample beam and of the reference beam, respectively. While several types of variable spiral plates (VSPs) could be used for generating OAM-carrying beams, the geometric phase elements can provide a direct connection between the SAM and OAM which serves as a spin–orbit-converter. In our design, the laser cavity is equipped with a Q-plate ($\rm{q}=+1/2$)^20^ to transform the left and right circular polarizations states ($|\rm{L}\rangle$ and $|\rm{R}\rangle$, respectively) to the eigenstates HOP_SS_ with opposite SAM and OAM ($\pm 2\rm{q\hslash }$), denoted by: $\left|+1,\rm{R}\right.\rangle$ and $|-1,\rm{L}\rangle$, respectively.

Dispersion is an inherent dilemma in femtosecond pulses with wide spectral bandwidth. It occurs in not only the spectral and spatial phase but also the geometric phase, which results in light of different wavelengths catches different OAM and polarization distribution. Therefore, we describe the output states by using a *mean* HOP sphere (${\bar{\mathbf{S}}}^{+1}$) which corresponds to the center wavelength (${\lambda }_{0}$). The north $(\Phi ,\uppi /2)$ and south $(\Phi ,-\uppi /2)$ poles represent the two eigenstates carrying spiral wavefronts with topological charge of 1 ($|(\Phi ,\uppi /2)\rangle =|-1,\rm{L}\rangle$ and $|(\Phi ,-\uppi /2)\rangle =|+1,\rm{R}\rangle$, respectively). In addition, the expressions for the radial and azimuthal polarization carrying states are $|(\rm{0,0})\rangle$ and $|(\uppi ,0)\rangle$, respectively. By modulating the angles between the x-axis of the laboratory system (*x*) and the optical axis of quarter wave plate III (QWP-III), $\rm{\alpha }$, and between *x* and that of the Q-plate, $\upbeta$, the input horizontal polarization states are mapped onto the entire *mean* HOP sphere ${\bar{\mathbf{S}}}^{+1}[(\Phi (\rm{\alpha },\upbeta ),\Theta (\rm{\alpha },\upbeta ))]$, where Φ and Θ are the polar and azimuthal angles of the spherical coordinates, respectively. The relationship between Φ, Θ and α, β is given by: $\Phi (\rm{\alpha },\upbeta )=2(\rm{\alpha }-\upbeta )$ and $\Theta \left(\rm{\alpha },\upbeta \right)=2\upbeta$, where $\rm{\alpha }\in \left[0, 2\uppi \right),\upbeta \in [\rm{0,2\pi })$. Details are described in the supplementary materials.

### Conventional pulse characterization

**Figure 2:**
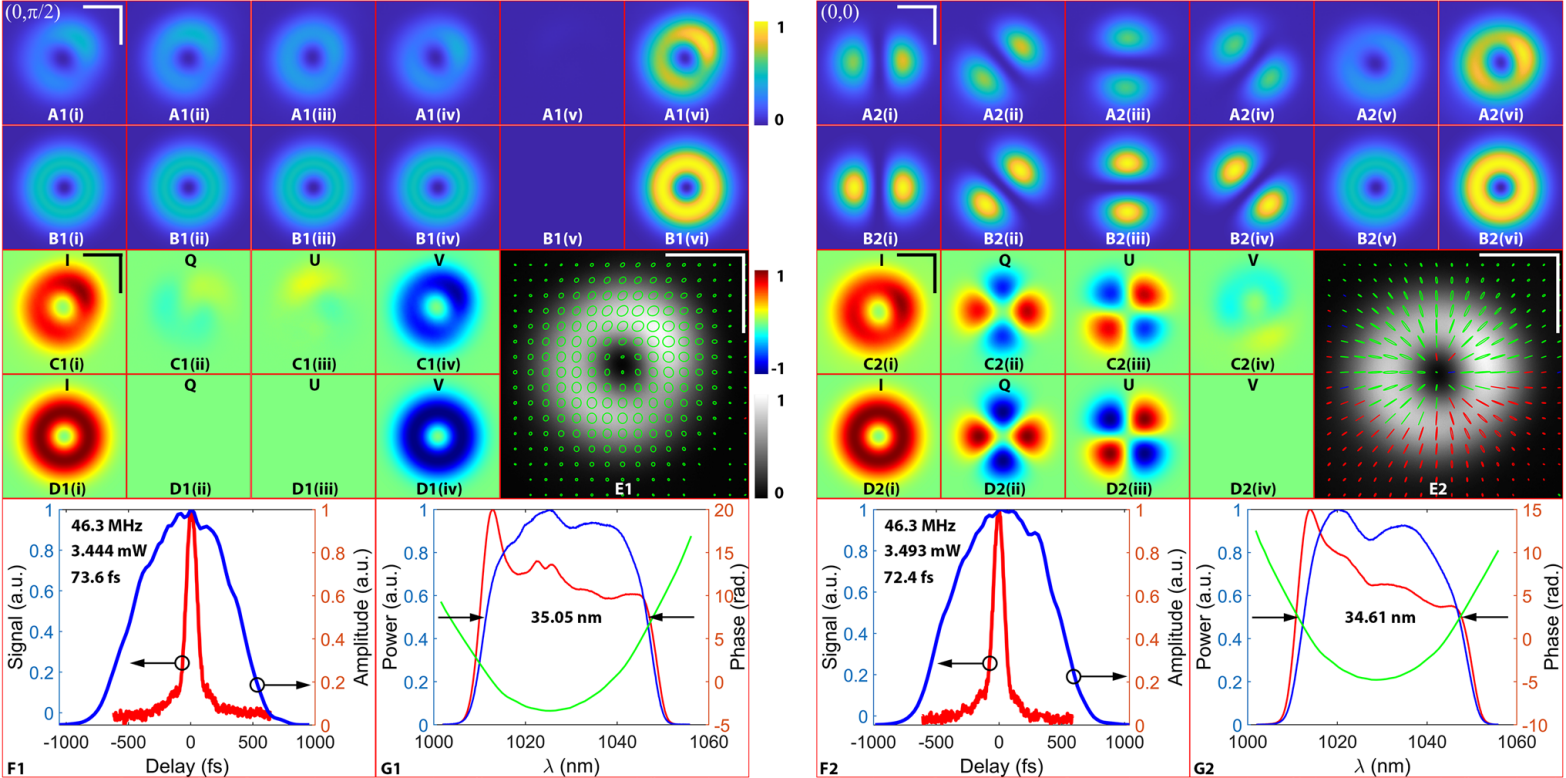
Conventional characterizations of the north pole and the radial states. A1-G1 (left): $\left|-1,L\right.\rangle$, the north pole $\left(0,\pi /2\right)$; A2-G2 (right): $(\left|-1,L\right.\rangle +\left|+1,R\right.\rangle )/\sqrt{2}$, the radial state $\left(0,0\right)$; (**A1(i–v))**: the 2D intensity measurements with different configurations; (**A1(i–iv))**: the patterns when the polarizer with orientation at: (i) = 0°; (ii) = 45°; (iii) = 90°; (iv) = 135°; (**A1(v))**: the pattern when the QWP and the polarizer are both orientated at 45°; (**A1(vi))**: the total intensity distribution; (**B1(i–vi))**: the corresponding simulations of A1; (**C1(i–iv))**: the Stokes parameters: (i) = I; (ii) = Q; (iii) = U; (iv) = V; **D1(i–iv)**: the corresponding simulations of C1; (**E1)**: the measured polarization ellipses, where green line denotes left-handed polarization, as red to right-handed polarization, and blue to linear polarization; (**F1)** is the pulse duration measurement of the reference beam retrieved by using the autocorrelator (red line) and FROG (blue line). The inserted numbers are, repetition rate, average output power of the sample pulse, and the pulse duration of the reference pulse with the assumption that the dechirped pulse carries Gaussian shape, respectively; (**G1)** is the spectrum of the sample beam (blue line), reference beam (red line), as well as the phase (green line) (The inserted numbers is the FWHM of the blue line). (**A2-G2)** follow the same descriptions. Scale bars represent 1 mm.

Figure 2 shows the conventional characterization of the intracavity-controlled OAM-carrying femtosecond laser with two common states on the HOP sphere: north pole $(0,+\uppi /2)$ (Left) and the radial $(\rm{0,0})$ (Right). A1(i–v) demonstrate the 2D intensity measurements with different configurations. A1(i–iv): the patterns when the polarizer with orientation at: (i) = 0°; (ii) = 45°; (iii) = 90°; (iv) = 135°; A1(v): the pattern when the QWP and the polarizer are both orientated at 45°; A1(vi): the total intensity distribution. B1(i–vi) are the corresponding simulations of A1. The simulations are based on the theory of the first-order vector beams^51^. C1(i–iv) show the Stokes parameters: (i) = I; (ii) = Q; (iii) = U; (iv) = V (Principles for the Stokes parameters measurements are detailed in the Supplementary Material: I.3). D1(i–iv): the corresponding simulations of C1. E1 is the measured polarization ellipses, where green line denotes left-handed polarization, as red to right-handed polarization, and blue to linear polarization. F1 is the pulse duration measurement of the reference beam retrieved by using the autocorrelator (red line) and FROG (blue line). The inserted numbers are, repetition rate, average output power of the sample pulse, and the pulse duration of the reference pulse with the assumption that the dechirped pulse carries Gaussian shape, respectively. G1 is the spectrum of the sample beam (blue line), reference beam (red line), as well as the phase (green line) (The inserted number is the FWHM of the blue line.). A2-G2 follow the same descriptions. Scale bars represent 1 mm. For the 2D spatial domain measurements on the cross-section, the experimental results and the simulations are well-matched, but not entirely. Firstly, the output modes (the total intensity distributions, Fig. 2A1(vi) and A2(vi)) are not the standard ${\mathbf{L}\mathbf{G}}_{0}^{\pm 1}$ mode (Fig. 2B1(vi) and B2(vi)) but distorted donut-like patterns; since the major axis of the intensity elliptical annulus rotates with the optical axis of the Q-plate, the intensity distributions on these annuli are not centrosymmetric about the center of the circle but rather concentrated on the two poles due to alignment imperfection. Secondly, the output polarization states are not pure. For example, in Fig. 2C1(ii) and C1(iii), the Q and U components of the north pole state are nonzero, indicative of residual linear polarization components in the output light. Impurities within the polarization states are further visualized by the north pole and radial polarization ellipses presented in Fig. 2E1 and E2, respectively. In the north pole state (Fig. 2E1), only a few perfect left-handed circular polarizations exist, and the axes of the left-handed elliptical polarization are modulated by some linear polarization components. The radial state shows cases of deteriorated linear polarization states with circular components (Fig. 2E2). Therefore, the purity of both the mode and polarizations should be further improved to produce the ideal, extremely refined output pulses. Measurements for other states, including south pole, azimuthal, $(\pi /2,\pi /4)$, $(0,-\pi /4),$ $(\pi /2, 0)$ and $(3\pi /2, 0)$ are reported in the supplementary materials.

For the single-pixel time and spectral domain measurements, the repetition rate of the laser is ~ 46 MHz. The temporal duration is ~ 70 fs (with the assumption that the dechirped pulse carries Gaussian shape), and the full width at half maximum (FWHM) of the spectrum is 34–36 nm (the Flip Mirror S (Fig. 1) is used to deflect the sample beam to the optical spectrum analyzer). The average output power is approximately 3–5 mW. The temporal duration and spectral phase were measured from the reference beam. The spectral phase measurement is necessary for polarization-sensitive spatiotemporal pulse characterization, which will be discussed in the next section. Although not comprehensively, these measurements still reveal some of the important pulse properties especially if the output states are continuously and adiabatically modulated along the HOP sphere.

**Figure 3:**
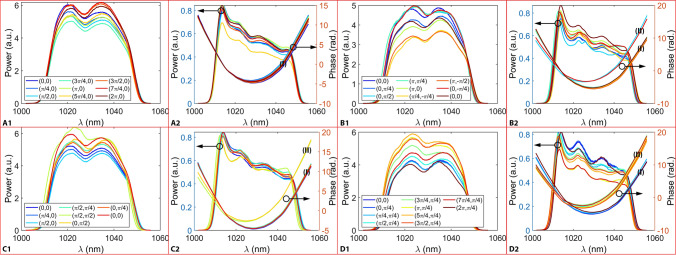
Spectrum and phase measurements of the pulse continually modulated along a closed path on the HOP sphere. (**A)**: the equator (Path A); (**A1)**: spectrum of sample pulse; (**A2)**: spectrum and phase of reference pulse; (**B)**: the circle of 0° and 180° longitude (Path B); (**C)**: the polar triangle between (0,0), (π/2,0) and (0,π/2) (Path C); D: the circle of 45° latitude (Path D). (**B–D)** follow the same description as A. Every specific spherical coordinate ($\Phi ,\Theta$) in the figure legend denotes a measurement point. I and II denote the two spectral phase zones containing the radial and the north pole state, respectively.

**Figure 4:**
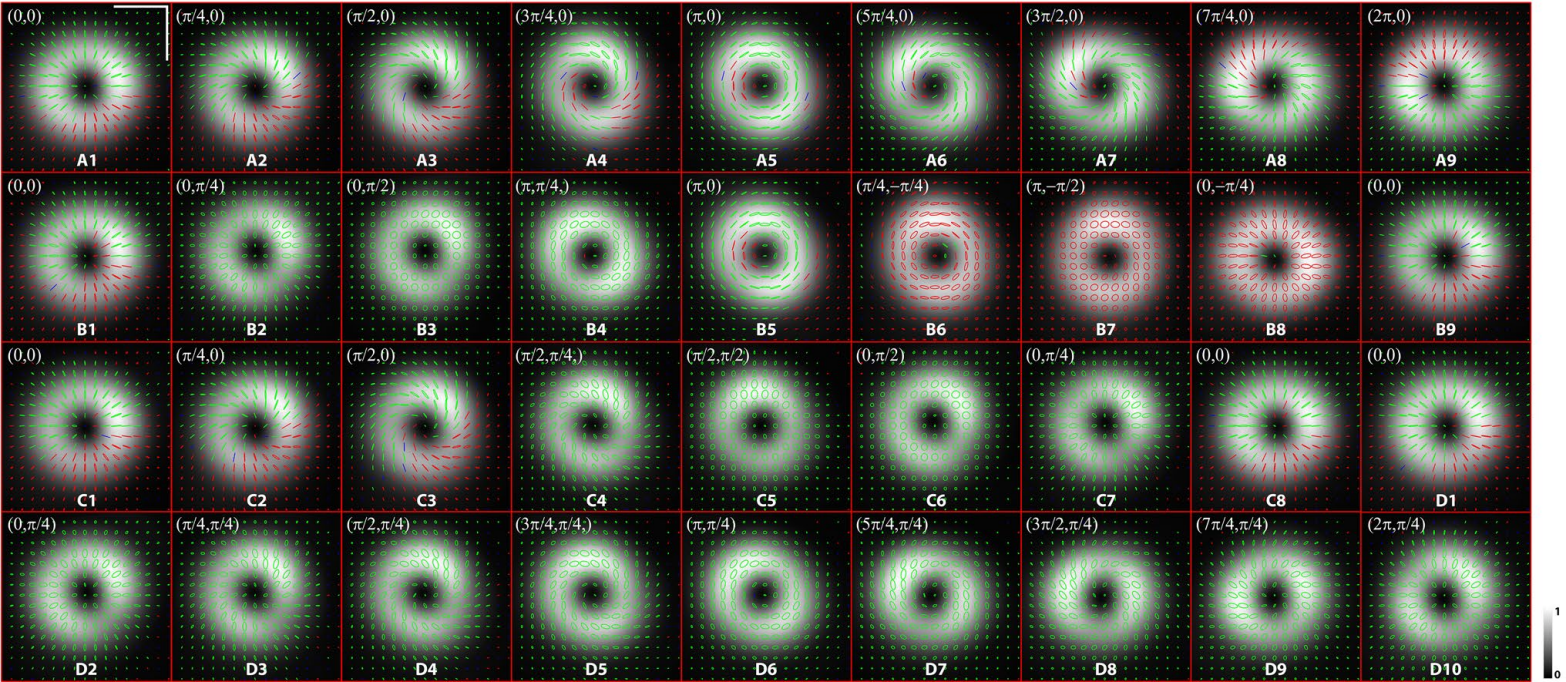
The polarization ellipses corresponding to Fig. 3. (**A1-9)**: Path A; (**B1-9)**: Path B; (**C1-8)**: Path C; (**D1-10)**: Path D. Each number represents one measurement point. All paths use the radial point state as the starting point. Scale bar represents 1 mm.

The abilities of continuous intracavity modulation of HOP_SS_ were accompanied by the alternations of the spectrum and the spectral phase due to the internal loss of the cavity. We demonstrate these in Fig. 3 by continually switching the state along a certain closed path, defined as the following: Path A, the equator; Path B, the circle of 0° and 180° longitude; Path C, the polar triangle between $(\rm{0,0})$, $(\pi /\rm{2,0})$ and $(0,\pi /2)$; Path D, the circle of 45° latitude, and; Path E, the circle of − 45° latitude (measurements of Path E are reported in the supplementary materials). Our measurements show that the spectral phases of 45 different states uniformly distributed on the sphere fall into two zones: Zones I and II, which include the radial and the north pole state, respectively. The polarization ellipses corresponding to the 4 Paths are presented in Fig. 4 to further demonstrate the pulse polarization structures as well as the capability of the femtosecond laser to generate a variety of HOP_SS_ beams.

### Characterization of pulse multidimensionality

The reference and sample spectra right after the PBS should be same. However, the QWP-III, VSP, and output coupler are spectral sensitive elements that will modulate the spectrum of the light passing through them. So, the final measured reference spectra are usually wider than its counterpart. Consequently, every spectral component of the sample pulse can be resolved through the interferometric pattern and the reference beam is well applicable for the multidimensional characterization. A plane wave is utilized as the reference beam as it produces an uniform interference fringe background, implying a constant spatial demodulation resolution. With an uniform linear polarization distribution, it can also provide measurement of one of the two components in a 2D vector field for the sample electric field across the reference beam wavefront. When the detection plane of the CCD, denoted by Plane ${\varvec{\Pi}}$, coincides with this wavefront, the projection of the sample electric field—which is essentially 2D vector field—on Plane ${\varvec{\Pi}}$ can be used to characterize the states of the pulse with good approximation because the amplitude of *z’* component of the electric field (mainly governed by $\Delta {\Omega }_{0}$, the angle between the reference and the sample beam) is only approximately one percent of the *x’* or *y’* component in this experiment. As such, by providing a homogenous reference beam across the whole field of Plane ${\varvec{\Pi}}$ and gaining the spectrum, the spectral phase data and the pulse duration via an optical spectrum analyzer, FROG^52^ and an interferometric autocorrelator (using the Flip Mirror R (Fig. 1) to deflect the reference beam to these instruments), the complete spectral domain information of the sample pulse can be obtained by using Equation S12 (detailed principle for polarization-sensitivity characterization described in the supplementary materials). We then can further reconstruct the temporal structure of the pulse based on Equation S13 after an inverse Fourier transform.

**Figure 5:**
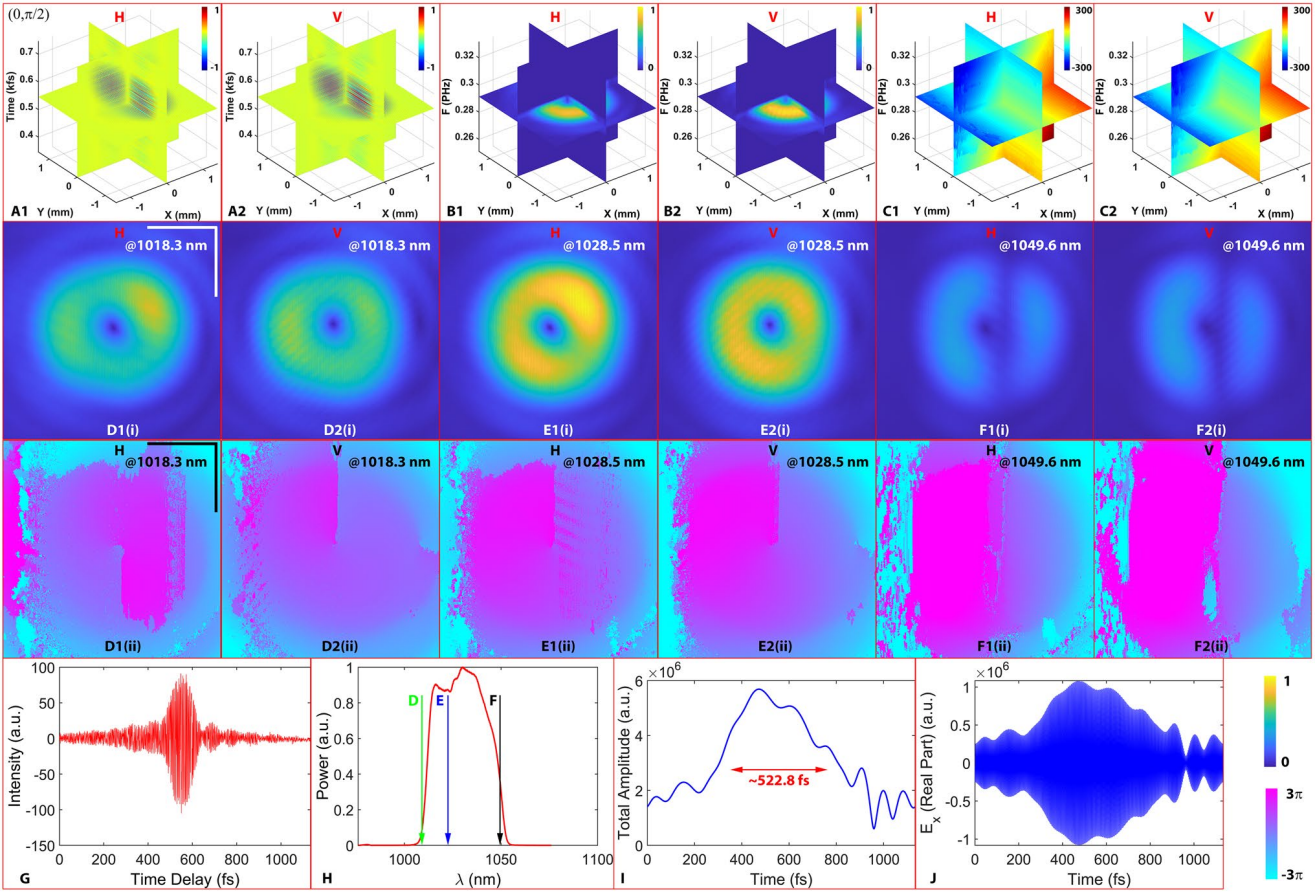
The polarization-sensitive spatiotemporal characterizations of the pulse with north pole state. (**A1-2)**: the 3D interferograms of the horizontal (*H*) and vertical (*V*) channels, respectively; (**B1-2)**: the corresponding 3D spectral amplitude of the two channels; (**C1-2)**: the corresponding 3D spectral phase. (**D)**, (**E)**, and (**F)**: the 2D amplitude (Roman numeral i) and unwrapped phase profiles (Roman numeral ii) at 1018.3 nm, 1028.5 nm and 1049.6 nm, respectively. (**G)**: single-pixel cross-correlation signal obtained from the time scanning procedure for *H* channel; (**H)**: the single-pixel spectrum; (**I)**: the single-pixel profile for the total temporal amplitude envelope; (**J)**: the corresponding real part of the pulse. Scale bars represent 1 mm.

**Figure 6:**
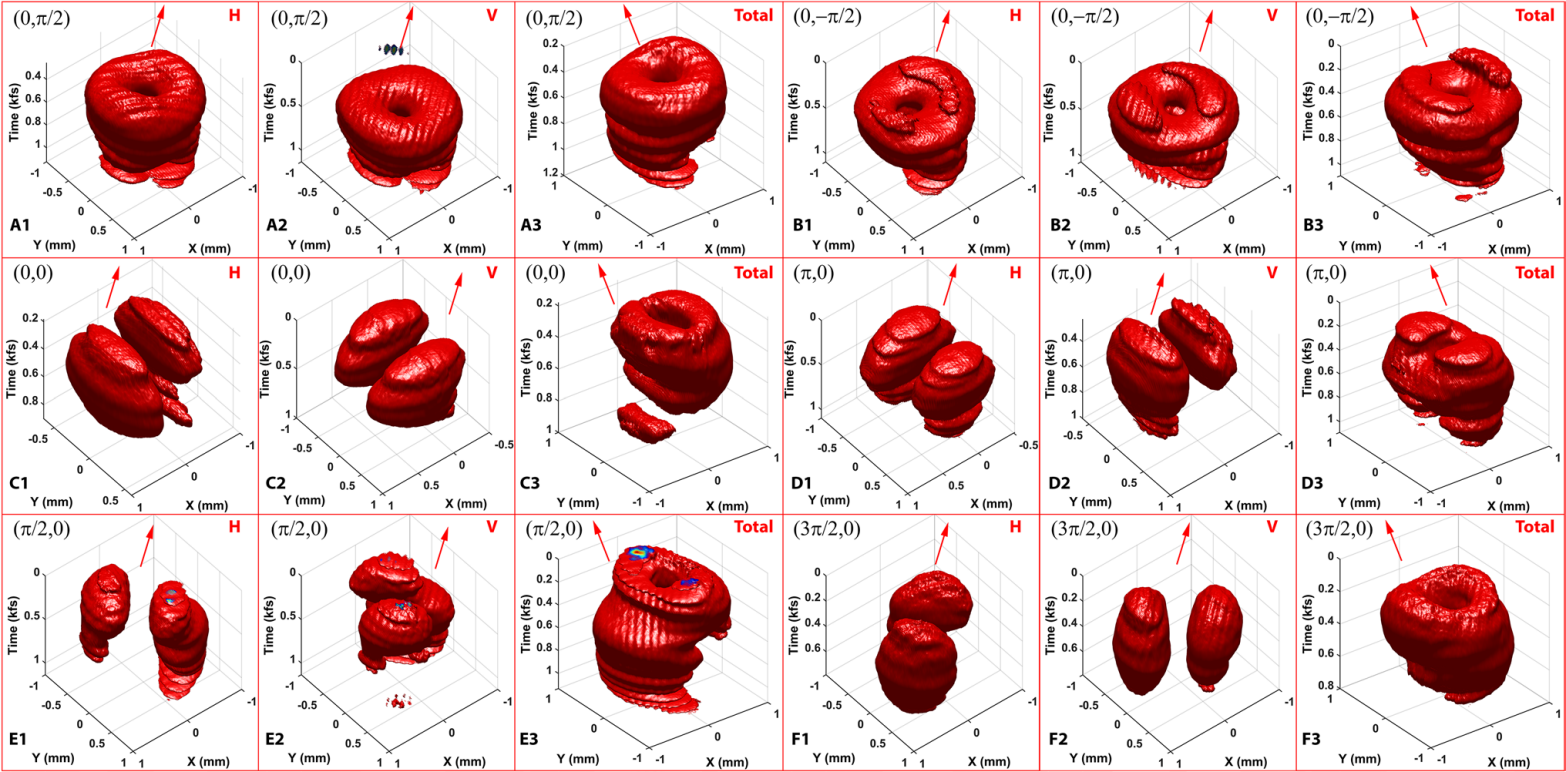
The 3D spatiotemporal reconstruction of pulse electric field ${\varvec{E}}({\varvec{r}},t)$ of six typical HOP_SS_. (**A)**: the north pole state; (**A1**) and (**A2**): amplitudes from the H and V channels, respectively; (**A3)**: the total amplitude; **B**: the south pole state; (**C**): the radial state; (**D)**: the azimuthal state; (**E)**: the state at $(\uppi /2, 0)$; (**F)**: the state at $(3\uppi /2, 0)$. (**B–F)** follow the same description as A. The isosurface (red surface) shown is set at 0.4 of the maximum amplitude.

Figure 5 demonstrates the polarization-sensitive spatiotemporal characterizations of the femtosecond pulse with the north pole state. Figure 5G shows a typical single-pixel cross-correlation signal obtained from the time scanning procedure. Figure 5A1-2 are the 3D interferograms recorded with the 2D time scanning for the horizontal (o’x’ axis) channel (Video S1), *H*, and the vertical (o’y’ axis) channel (Video S2), *V*, respectively. Due to tilted sectioning and the donut-shaped pulse feature, the 2D interferograms provide inhomogeneous intensity distribution. Figure 5B1-2 are the corresponding 3D spectral amplitudes of the two channels after applying a Fourier transform and a super-Gaussian spectral filter. Figure 5C1-2 present the 3D spectral phases, revealing the complexity of the structural phase information. The X and Y axes in Fig. 5A–F coincide with *x’* and *y’*, respectively. Collectively, the measurements shown in Fig. 5 illustrate both the global (donut-shaped spectral amplitude) and local (spiral spatial-spectral phase) spectral structures of the complex pulse which is essentially equivalent to the corresponding temporal structures (discussed later in Fig. 6). The single-pixel spectrum at different spatial points reveals microscopic differences within one channel, and even more drastic between the two channels, which is an evidence for the spatiotemporal coupling effects in the spectral domain^48^ as well as the polarization structures. In addition, the dominant background of the 3D phase structure is the global phase ($2\pi x{^{\prime}}\rm{tan}\left(\Delta {\Omega }_{0}\right)/\lambda$, $\lambda$ is the picked-up wavelength.) generated by $\Delta {\Omega }_{0}$, which can be removed to uncover the local phase structure, such as the spiral wavefront. Applying the background clearing operation by subtracting out a set of specific frequency-dependent phases on Plane ${\varvec{\Pi}}$ at three different frequencies (1018.3 nm, 1028.5 nm and 1049.6 nm, Fig. 5H), and the resulting 2D amplitude and the corresponding unwrapped phase profiles are shown in Fig. 5D–F (amplitude shown in (i), and unwrapped phase profiles shown in (ii)). The amplitude profiles from both *H* and *V* channels at all three wavelengths exhibit the donut-shaped contours that are well-matched with the 3D amplitude structures, and the unwrapped phase precisely demonstrates the spiral feature of the vortex beam carrying the helicity of − 1. Figure 5I is the single-pixel signal of the temporal amplitude of the chirped pulse with the measured FWHM of ~ 500 fs, and Fig. 5J is the corresponding real part of the *H* channel, which includes 8192 sampling points in order to resolve the carrying frequency.

The time scanning procedure allows for reconstruction of the pulse electric field, ${\varvec{E}}({\varvec{r}},t)$, in the spatiotemporal domain. The electric field (i.e., the total amplitude) is the root mean square of the two channels and is insensitive to the relative phase. Figure 6 shows the 3D reconstructions of the north pole (Videos S3-5), south pole (Videos S6-8), radial (Videos S9-11), azimuthal (Videos S12-14), $(\pi /2, 0)$ (Videos S15-17), and $(3\pi /2 ,0)$ (Videos S18-20) of the HOPss. The red arrows denote the propagation direction of the pulse, ${{\varvec{k}}}_{0}$, defined by a calibration pulse with a plane wavefront, which can be realized by removing the Q-plate in the laser cavity. The temporal axis, $t$ (Time axis in Fig. 6), is antiparallel with the *z’* axis as $\varphi ={\omega }_{0}t-{{\varvec{k}}}_{0}\bullet {\varvec{r}}$, where $\varphi$ is the phase and ${\omega }_{0}$ is the center angular frequency of the pulse. As shown in Fig. 6A–B, the north pole and south pole states temporally maintain a donut-like 3D shape in both *H* and *V* channels as well as the total amplitude structure, supported by the conventional characterization method shown in Fig. 2A1-A2 (*H* channel shown in (i), *V* channel shown in (iii), and total amplitude shown in (vi)). This demonstrates the accumulation effects of the pulses injecting onto Plane ${\varvec{\Pi}}$. Although bearing a similar amplitude structure, minor discrepancies in both the spatial domain (the X–Y plane) and the temporal domain (the Time axis) between the two states are still noted. These discrepancies are caused by the diversity between the spectral phase shown in Fig. 2G1–G2. Figure 6C and F depict the results from additional four states along the equator, including $(\rm{0,0})$, $(\pi /\rm{2,0})$, $(\pi ,0)$, and $(3\pi /\rm{2,0})$. The total amplitude profiles of these states show the donut-shaped structure as anticipated. In addition, the local profile of the two sub-pulses, whose axis of symmetry rotates with respect to the state being modulated, can be visualized through the *H* and *V* channels. However, the spectral bandwidth of the output pulses is inadequate to generate a distinct twisting effect of the polarization distributions along the temporal axis—which is caused by the dispersion of the VSP—that provides different Pancharatnam-Berry phase for different wavelengths. An ultra-wideband pulse or a large geometric phase dispersive device can be utilized to visualize the twisting effect, such as a pulse temporally transformed from radial to azimuthal polarization.

In our multidimensional analysis of pulses, all total amplitude profiles affirm a donut-shaped pulse that is supported by the 2D spatial structure prediction. Nevertheless, the intrinsic spectral phase will decidedly modify the temporal structure, as shown in Fig. 6A and C. The polarization resolvability of the system is further verified by the different spatiotemporal structures from the *H* and *V* channels within the measured HOP_SS_ pulse. For instance, Fig. 6C–F demonstrate that specific spatial features (lobe structures in the *H* and *V* channels) of a pulse are determined by its vectorial properties, and that the temporally accumulated pattern is consistent with the 2D conventional measurement. It is worth noting that the system can resolve any tilted input sample light fields as long as the angle between the signal propagation direction and the normal of Plane ${\varvec{\Pi}}$ is within ± 3.18°. Detailed descriptions are reported in the supplementary materials.

## Discussion

We have presented a femtosecond pulse scope that unites vector vortex mode-locked lasing and vectorial quantification. By controlling the Pancharatnam-Berry phase via the VSP and QWP within the laser cavity, OAM-carrying beams (i.e., HOP_SS_) can be produced and continually modulated while imposing minimal effect on the status of the passive mode-locked operation. Most of the laser configurations described above are self-start. However, it is generally more favorable to achieve a given state through self-starting at the radial state $(0, 0)$ then continuously modulating to the target state. Given a diode-pumped light with adequate power, the mode-locked operation remains stable for weeks. The presence of the free-space section does not degrade the long-term stability of the laser. In practice, the intracavity configuration can be conveniently adapted into existing mode-locked lasers, enabling a flexible tailoring of the structured light in spatial, temporal, and spectral domains, as well as polarization distribution. With these unique features, this pulse-scope system can provide an unique light source for spatiotemporal nonlinear optics, laser machining, nanoparticle manipulation, and spatial mode division multiplexed systems.

Laser pulses in either laboratory or industry are typically complex objects. Unlike the classical electromagnetic (EM) wave with uniform polarization distribution along a flat wavefront or a general vector beam under the paraxial approximation, real-world light pulses, such as beams from a high-power multimode fiber laser, dechirped femtosecond pulses with structured features, or even more intricate optical phenomena (e.g., knot-shaped pulses), usually carry a non-vanishing component in the propagation direction. Therefore, the description of a general vectorial laser pulse should ideally be implemented in a multidimensional way. Herein, we have introduced the initial concept and implementation of a polarization-sensitive temporal scanning interferometer to capture the sophisticated information of these complex HOP_SS_ pulses. The pulse-scope system provides the information on spatiotemporal amplitude and phase distribution as well as the polarization-sensitive features of the complex laser pulses.

Like most laser systems, mechanical instability and environmental vibrations may degrade the measurement accuracy. The pulse scope characterizes the beam without the relative phase between the *H* and *V* channels; the capability of recording null relative phase information can further enable spatiotemporal reconstruction of a complex pulse as a 3D vector field. The quantification ability of this pulse-scope system, nevertheless, assures a new means of exploration for in-depth characterization and optimization of ultrafast laser pulse, paving the way for investigating novel pulse generations as well as for studying the interactions between materials and structured pulses. This integrated device will facilitate adaptive customization of structured pulses that can be applied in optical physics research and laser-based manufacturing.

## Material and methods

### Intracavity-controlled OAM-carrying femtosecond laser

The laser system (gray portion of the floor, Fig. 1) has an unidirectional ring cavity employed for self-starting operation, in which the propagation direction is denoted by the red circular arrow. The gain medium, a 26-cm long Ytterbium-doped fiber, is pumped by a low-profile laser diode which can deliver up to 500 mW (at 980 nm) fiber-coupled kink free output power through a 980/1030-nm wavelength-division multiplexing coupler. Passing through QWP-I, the polarizing beamsplitter (PBS, extinction ratio of 1000:1), QWP-III, and the VSP, the light launched from the transmitting collimator (collimator-Tx) is reflected back to the cavity by the output coupler with reflectivity of 90% and the unprotected gold mirror II (Mirror-II). Two pairs of reflective diffraction gratings (600 lines/mm, 1-μm blaze) are used to compensate for the normal group-velocity dispersion of the fiber. The PBS—which maintains its p-polarization along *x*—QWP-III, the VSP, the output coupler, Mirror-II, and half wave plate I (HWP-I) composes a HOP sphere beams generator that produce the on-demand HOP_SS_ from the pure, horizontal (*x*) linear polarization states. From the output coupler, the reflected light then transforms its polarization distribution back to the linear polarization state which can be expressed as $-\rm{cos}(2\upbeta ) {\widehat{\mathbf{e}}}_{\mathbf{x}}+\rm{sin}(2\upbeta ){\widehat{\mathbf{e}}}_{\mathbf{y}}$; that is, the polarization direction has a degree of $2\upbeta$ over *x*, assuming the angle between the input and reflected light (~ 0.85°) is negligible (detailed in the supplementary materials). Here, HWP-II is employed to rotate the polarization of the reflected light from $2\upbeta$ to 0 to further reduce the additional loss of the whole cavity because it is crucial to ensure that the vibrational direction of the light launched to the reflected diffraction grating is perpendicular to the direction of the groove due to the polarization-sensitive property of the grating. The PBS also provides the reference plane wave which carries the vertical (*y*) linear polarization for the polarization-sensitive spatiotemporal pulse characterization system. The light then passes through the isolator, HWP-I, QWP-I before collected by the receiving collimator (collimator-Rx) into the 130-cm single-mode fiber (SMF). Mode-locked operation is stabilized by nonlinear polarization evolution which is implemented with the bulk wave plates (HWP-I, QWP-I and QWP-II) and SMF.

### Conventional pulse characterization

To identify the pulse duration of the reference beam (pink arrow, Fig. 1), we assumed the reference beam is transversely homogeneous and thus has a well-defined pulse duration relative to the complex sample pulse. Thus, a custom-built interferometric autocorrelator equipped with a two-photon photodetector is used to measure the reference beam pulse duration.

The conventional measurement of the polarization distribution was realized through a 2D imaging polarimeter, which consists of a complementary metal–oxide–semiconductor (CMOS) camera, a polarizer, and a QWP. The reference frame is calibrated by a single-point, free-space polarimeter system. The measurement procedure is standard, which is detailed in the supplementary materials. The spatial distribution of the Stokes parameters or polarization ellipses only describes the average vectorial properties of the pulse on the cross section, and cannot provide the temporal information. From the spectral domain perspective, these measured Stoke parameters are the weighted summation of all common Stokes parameters corresponding to a single wavelength.

### Characterization of pulse multidimensionality

In the multidimensional analysis, a 2D polarization-sensitive temporal scanning Mach–Zehnder interferometer (green portion of the floor, Fig. 1) was used to capture the complete information of the pulses. The reference beam, exported from the PBS, is first time-delayed via an unprotected gold right-angle prism mirror, an unprotected gold hollow roof prism mirror, and a linear stage, then attenuated to a suitable intensity by a round variable reflective neutral density filter (NDF-I). Thereafter, the beam passes through a flat optical window (OW-I) for precisely balancing the dispersion between the two arms, and the polarizer rotates automatically by a motorized rotation stage. The reference beam is then expended by a laboratory-made 10 × beam expender consisting of Lens-I (f = 7.5 mm), Lens-II (f = 75 mm), and a 5-μm pinhole, which selects a small portion of the energy of the reference beam around the focus, before reaching the beamsplitter (BS). The sample beam is launched from the output coupler, and then propagates through NDF-II, OW-II, Mirror-IV and -V, and finally interferes with the reference beam at the BS. Mirror-IV and -V are used to compensate for the extra optical pathlength due to the delay line in the reference arm. A CCD with a sensor size of 1034 × 768 pixels and an unit cell size of 4.65 × 4.65 μm sequentially captures the 2D interference patterns. Temporal scanning, containing 1,700 steps, is achieved by discretely translating the hollow roof prism on the linear stage. The scanning step size of 100 nm corresponds to the optical path delay $(\delta z)$ of 200 nm due to the double path configuration (the right-angle prism and the hollow roof prism), equivalent to a spectral detection range $(\Delta \lambda )$ of 5.3 μm. The spectral frequency resolution $(\delta \lambda )$ is 3.1 nm calculated using the whole delay range $(\Delta z)$ of 340 μm, and the spatial resolution of the system $(\delta x)$ of 37.2 μm accords with $\Delta {\Omega }_{0}$ of ~ 1.6°. The maximum resolvable angle $(\Delta {\Omega }_{max})$ is 6.36°, determined by the pixel size of the CCD and Nyquist–Shannon sampling theorem. Generally, the EM fields can be represented by a superposition of plane waves; that is, the complex pulse is the combination of plane waves carrying different propagation directions (wave vector ${\varvec{k}}$) but with small differences under the paraxial approximation. Therefore, this system meets the requirement for resolving the sample complex pulses and even scattered light, in which the small scattering angle carrying forward scattered light are the dominant portion. Even though the 2D polarization-sensitive temporal scanning Mach–Zehnder interferometer is a powerful tool to resolve the complete structure of the ultrafast pulses, when applying it to the few-cycle pulses in the near-infrared wavelength that has significantly larger bandwidth some important factors should be carefully considered: (1) the inadequate spectral responsivity of the CCD camera will consequently set a limited resolvable spectrum bandwidth for the whole system; (2) the fluctuation in spectrum, propagation direction, and wavefront on the input pulses have significant effects on the spatiotemporal resolution and contrast of the interferograms; (3) since mechanical scanning steps are not perfect, fluctuations in the delay above a certain threshold will cause a degradation of the retrieved spectrum.

## Supplementary Information

Supplementary Information 1.

Supplementary Information 2.

Supplementary Information 3.

Supplementary Information 4.

Supplementary Information 5.

Supplementary Video 1.

Supplementary Video 2.

Supplementary Video 3.

Supplementary Video 4.

Supplementary Video 5.

Supplementary Video 6.

Supplementary Video 7.

Supplementary Video 8.

Supplementary Video 9.

Supplementary Video 10.

Supplementary Video 11.

Supplementary Video 12.

Supplementary Video 13.

Supplementary Video 14.

Supplementary Video 15.

Supplementary Video 16.

Supplementary Video 17.

Supplementary Video 18.

Supplementary Video 19.

Supplementary Video 20.
