# Supplementary material for: Integrated pulse scope for tunable generation and intrinsic characterization of structured femtosecond laser
Source: Sci Rep. 2021 May 6;11:9670. doi: 10.1038/s41598-021-87938-w (PMC8102529; doi:10.1038/s41598-021-87938-w)
Supplement: Supplementary file 1 — Supplementary Information 1. [file 41598_2021_87938_MOESM1_ESM.docx]

**Supplementary Material: Integrated pulse scope for tunable generation and intrinsic characterization of structured femtosecond laser**

Tiancheng Huo, Li Qi, Jason J. Chen, Yusi Miao and Zhongping Chen

Beckman Laser Institute and Medical Clinic, Department of Biomedical Engineering, University of California, Irvine, Irvine, CA 92612, USA

In this supplementary information, we provide detailed descriptions of this integrated system for intracavity generation and multidimensional characterization of broadband HOP_SS_ femtosecond pulses, and present validation tests of the measurement results. This document is organized as follows:

In Section I, we describe the calculations for the laser cavity set-up and the method for the Stokes parameters measurements as well as its data processing.

In Section II, we detail the numerical processing technique applied to the raw experimental data to obtain the spatiospectral and spatiotemporal properties of the femtosecond pulses.

**Section I: Calculations for the laser cavity setup and method for the Stokes parameters measurements as well as its data processing**

**I.1. Calculating the map between the HOP_SS_ and the parameter** $\boldsymbol{\alpha}$**,** $\boldsymbol{\beta}$

Geometric phase-based elements, such as Q-plates with different topological charge, J-plates, and sectored spatially varying retarders, can provide a direct connection between the SAM and OAM which serves as a spin-orbit-converter. This conversion depends on the symmetricity of the local optical axis distribution on the device, and it transforms a well-defined monochromatic plane electromagnetic (EM) wave with pure polarization states descripted by arbitrary points on a common Poincaré sphere to the states on the HOP sphere, where the two poles are generated by the two monochromatic plane EM wave with pure circular polarization states carrying opposite helicity (left and right circular polarization states: $|L\rangle$ and $|R\rangle$). Here, the laser is equipped with Q-plate ($q=+1/2$), the most common geometric phase device, to perform the transformation where the left and right circular polarizations are converted to output states with opposite spin and $\pm2q\hbar$ OAM, as follows:

|  | $\vert L\rangle\to\vert+2q,R\rangle=\vert+2q\rangle\otimes\vert R\rangle=exp(+i2q\varphi)\vert R\rangle$  $\vert R\rangle\to\vert-2q,L\rangle=\vert-2q\rangle\otimes\vert L\rangle=exp(-i2q\varphi)\vert L\rangle$ | **(S1)** |
| --- | --- | --- |

where $|\pm2q\rangle=exp(\pm i2q\varphi)$ is the azimuthal coordinate, and $\varphi=arctan(y/x)$.

By modulating the angles between the x-axis of the laboratory system (*x*) and the optical axis of QWP-III, α, and between *x* and that of the Q-plate, β, the input horizontal polarization states are mapped onto the entire mean HOP sphere $[{\bar{\mathbf{S}}}^{\mathbf{+1}}(\Phi(\alpha,\beta),\Theta(\alpha,\beta)$)], where $\Phi$ and $\Theta$ are the polar and azimuthal angles of the spherical coordinates, respectively.

Using the Jones calculus, the input state after the PBS can be the expressed as:

$$\left| \mathrm{Input} \right\rangle=E_{x0}\left( t \right)e^{i\left[ \varphi_{x0}\left( t \right)-\omega_{0}t \right]}{\hat{\mathbf{e}}}_{\mathbf{x}}=E_{x0}\left( t \right)e^{i\left[ \varphi_{x0}\left( t \right)-\omega_{0}t \right]}\left[ \begin{matrix} 1 \\ 0 \end{matrix} \right]$$

where $\omega_{0}$ is the central angular frequency, and $\varphi_{x0}\left( t \right)$ and $E_{x0}\left( t \right)$ are the phase and amplitude corresponding to the envelope, respectively. ${\hat{\mathbf{e}}}_{\mathbf{x}}$ is the unit vector of *x*-axis of the laboratory system (*oxyz*). Furthermore, the Q-plate and the QWP have the matrix formulation as:

$$M_{Q-plate}=\left[ \begin{matrix} cos(2q\varphi+2\alpha) & sin(2q\varphi+2\alpha) \\ sin(2q\varphi+2\alpha) & -cos(2q\varphi+2\alpha) \end{matrix} \right]$$

$$M_{QWP}=\left[ \begin{matrix} {cos}^{2}(\beta)+i{sin}^{2}(\beta) & {\sqrt{2}e}^{-i\frac{\pi}{4}}sin(\beta)cos(\beta) \\ {\sqrt{2}e}^{-i\frac{\pi}{4}}sin(\beta)cos(\beta) & {sin}^{2}(\beta)+i{cos}^{2}(\beta) \end{matrix} \right]$$

Therefore, the output states $\left| {\bar{\mathbf{S}}}^{\mathbf{+1}}(\Phi\left( \alpha,\beta\right),\Theta\left( \alpha,\beta\right) \right\rangle$ can be calculated as:

$$\left| {\bar{\mathbf{S}}}^{\mathbf{+1}} (\Phi\left( \alpha,\beta\right),\Theta\left( \alpha,\beta\right) \right\rangle={M_{Q-plate}M}_{QWP}\left| \mathrm{Input} \right\rangle={\frac{1}{\sqrt{2}}E}_{x0}\left( t \right)e^{i\left[ \varphi_{x0}\left( t \right)-\omega_{0}t \right]}\left[ \begin{matrix} e^{-i\frac{\pi}{4}}\cos\left( 2q\varphi-2\beta+2\alpha\right)+e^{+i\frac{\pi}{4}}\cos\left( 2q\varphi+2\alpha\right) \\ e^{-i\frac{\pi}{4}}\sin\left( 2q\varphi-2\beta+2\alpha\right)+e^{+i\frac{\pi}{4}}\sin\left( 2q\varphi+2\alpha\right) \end{matrix} \right]$$

In the picture of the circular polarization, this equation can be expressed as:

|  | $\left\vert{\bar{\mathbf{S}}}^{\mathbf{+1}}(\Phi\left( \alpha,\beta\right),\Theta\left( \alpha,\beta\right) \right\rangle=\sin(\frac{\Theta}{2}+\frac{\pi}{4})e^{-i\frac{\Phi}{2}}\left\vert-1,L \right\rangle+\cos(\frac{\Theta}{2}+\frac{\pi}{4})e^{+i\frac{\Phi}{2}}\left\vert+1,R \right\rangle$ | **(S2)** |
| --- | --- | --- |

where $\left| L \right\rangle$ and $|R\rangle$ have the following forms in the picture of the linear polarization:

$\left| L \right\rangle={\frac{1}{\sqrt{2}}E}_{x0}\left( t \right)e^{i\left[ \varphi_{x0}\left( t \right)-\omega_{0}t \right]}\left[ \begin{matrix} 1 \\ i \end{matrix} \right]$ and $\left| R \right\rangle={\frac{1}{\sqrt{2}}E}_{x0}\left( t \right)e^{i\left[ \varphi_{x0}\left( t \right)-\omega_{0}t \right]}\left[ \begin{matrix} 1 \\ -i \end{matrix} \right]$

The relationship between Φ, Θ and α, β is:

|  | $\Phi\left( \alpha,\beta\right)=2\left( \alpha-\beta\right),$  where $\Theta\left( \alpha,\beta\right)=2\beta$, and $\alpha\in\left[ 0, 2\pi\right), \beta\in[0,2\pi)$ | **(S3)** |
| --- | --- | --- |

**I.2. Calculation for the light reflected state from the output coupler**

With respect to reflected direction (reverse of the *oz* direction) of the femtosecond pulse, the Q-plate, the QWP and the output coupler have the matrix formulations shown as follow:

$$\bar{M}_{Q-plate}=\left[ \begin{matrix} cos(2q\varphi+2\alpha) & -sin(2q\varphi+2\alpha) \\ -sin(2q\varphi+2\alpha) & -cos(2q\varphi+2\alpha) \end{matrix} \right]$$

$$\bar{M}_{QWP}=\left[ \begin{matrix} {cos}^{2}(\beta)+i{sin}^{2}(\beta) & {-\sqrt{2}e}^{-i\frac{\pi}{4}}sin(\beta)cos(\beta) \\ {-\sqrt{2}e}^{-i\frac{\pi}{4}}sin(\beta)cos(\beta) & {sin}^{2}(\beta)+i{cos}^{2}(\beta) \end{matrix} \right]$$

$$\bar{M}_{output-coupler}=\left[ \begin{matrix} -1 & 0 \\ 0 & 1 \end{matrix} \right]$$

Therefore, the back-reflected signal can be calculated as:

|  | $\left\vert\mathrm{Reflected} \right\rangle={\bar{M}_{QWP}\bar{M}}_{Q-plate}\bar{M}_{output-coupler}\left\vert{\bar{\mathbf{S}}}^{\mathbf{+1}}(\Phi\left( \alpha,\beta\right),\Theta\left( \alpha,\beta\right) \right\rangle=\left[ \begin{matrix} -cos(2\beta) \\ sin(2\beta) \end{matrix} \right]$ | **(S4)** |
| --- | --- | --- |

which means the polarization direction has a degree of double β over *x* if one ignores the small angle between the input and reflected light (~0.85°).

**I.3. Principles for the Stokes parameters measurements**

If the monochromatic electric field is described as:

$$\boldsymbol{E}(\boldsymbol{r},t)=\left[ \begin{matrix} E_{x}(\boldsymbol{r},t) \\ E_{y}(\boldsymbol{r},t) \end{matrix} \right]=\left[ \begin{matrix} E_{x0}e^{-i(\omega t-\boldsymbol{k\cdot r}+\varphi_{x})} \\ E_{y0}e^{-i(\omega t-\boldsymbol{k\cdot r}+\varphi_{y})} \end{matrix} \right]$$

where $\omega$ is the angular frequency, $\boldsymbol{k}$ is the wave vector, $E_{x0}$ and $E_{y0}$ are the constant amplitude, and $\varphi_{x}$ and $\varphi_{y}$ are the constant phase, then the definition of the Stokes parameters ($I, Q, U,V$) is:

|  | $\left\{ \begin{aligned} \begin{matrix} I=E_{y0}^{2}+E_{x0}^{2} \\ Q=E_{y0}^{2}-E_{x0}^{2} \end{matrix} \\ \begin{matrix} U=2E_{y0}E_{x0}cos(\varphi_{yx}) \\ V=2E_{y0}E_{x0}sin(\varphi_{yx}) \end{matrix} \end{aligned} \right.$ | **(S5)** |
| --- | --- | --- |

where $\varphi_{yx}=\varphi_{y}-\varphi_{x}$.

The characterization system for the Stokes parameters includes three parts: the QWP, the linear polarizer, and a CCD, as shown in Figure S1. The measurement procedures are shown in the Table S1. First, a linear polarizer is placed in front of the CCD with its fast axis at 0° to record $E_{x0}^{2}$, then at 45° to record $\frac{1}{2}[E_{y0}^{2}+E_{x0}^{2}+2E_{y0}E_{x0}cos(\varphi_{yx})]$, at 90° to record the $E_{y0}^{2}$. This is followed by placing a QWP with its fast axis at 45° and the same linear polarizer at 45° in front of the CCD to obtain the signal $\frac{1}{2}[E_{y0}^{2}+E_{x0}^{2}-2E_{y0}E_{x0}sin(\varphi_{yx})]$.

**Figure S1.** Schematic diagram of the characterization system for the Stokes parameters. QWP: quarter-wave plate. The reference systems *oxyz* denotes the laboratory system, and the positive direction of *oz* refers to the propagation direction of the input pulses (denoted by the red arrow).


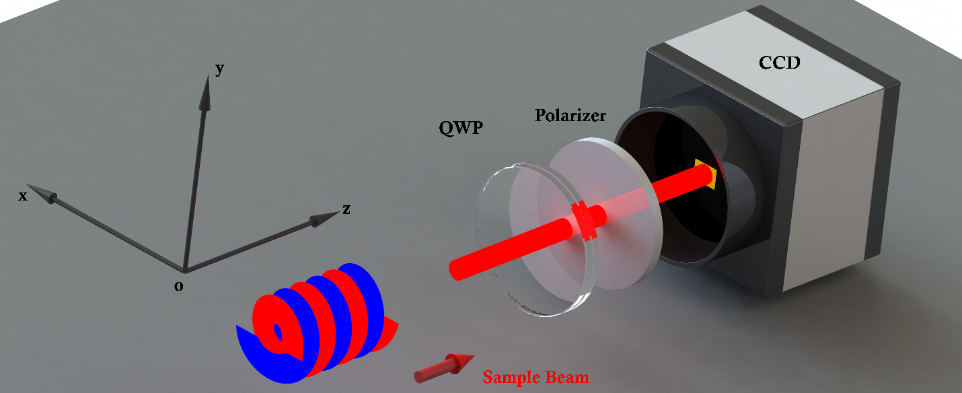


| **Step** | **Configuration** | **Measured Signal** | **Relationship between the measured signal and its amplitude and the phase** |
| --- | --- | --- | --- |
| 1 | Polarizer @ $0^{\circ}$ | $I_{0^{\circ}}$ | $I_{0^{\circ}}=E_{x0}^{2}$ |
| 2 | Polarizer @ 45$^{\circ}$ | $I_{45^{\circ}}$ | $I_{45^{\circ}}=\frac{1}{2}[E_{y0}^{2}+E_{x0}^{2}+2E_{y0}E_{x0}cos(\varphi_{yx})]$ |
| 3 | Polarizer @ 90$^{\circ}$ | $I_{90^{\circ}}$ | $I_{90^{\circ}}=E_{y0}^{2}$ |
| 4 | QWP @ 45$^{\circ}$ (before) + Polarizer @ 45$^{\circ}$ (after) | $I_{QP}$ | $I_{QP}=\frac{1}{2}[E_{y0}^{2}+E_{x0}^{2}-2E_{y0}E_{x0}sin(\varphi_{yx})]$ |

**Table S1.** The procedures for the Stokes parameters measurements.

Based on the table 2, the expression for the Stokes parameters can be obtain:

$\left\{ \begin{aligned} \begin{matrix} I=I_{90^{\circ}}+I_{0^{\circ}} \\ Q=I_{90^{\circ}}-I_{0^{\circ}} \end{matrix} \\ \begin{matrix} U={2I}_{45^{\circ}}-{(I}_{90^{\circ}}+I_{0^{\circ}}) \\ V={(I}_{90^{\circ}}+I_{0^{\circ}})-{2I}_{QP} \end{matrix} \end{aligned} \right.$ (S6)

Because the femtosecond pules are broadband signal, the Stokes parameters demonstrate the accumulated effects that the results are the summation of the different Stokes parameter corresponding to the different single wavelength.

**I.3. Conventional characterization of the intracavity-controlled OAM-carrying femtosecond laser**

In this section, more experimental results for the conventional characterization are presented. All results are grouped into five parts corresponding to the five modulation paths along the HOP sphere, which is shown in the Figure S2. The five paths include: Path A, the equator or the red circle; Path B, the circle of 0° and 180° longitude, or the green circle; Path C, the polar triangle between (0,0), (π/2,0) and (0,π/2); Path D, the circle of 45° latitude, or the blue circle with $\Theta=\pi/4$, and; Path E, the circle of −45° latitude, or the cyan circle with $\Theta=-\pi/4$. $\alpha$ and $\beta$ each has an offset of −8° and −4.9°, respectively.

**I.3.A. Path A, the Equator**

This part demonstrates the detailed characterizations for the states modulated along the path A. Table S2A shows the theoretical (denoted by the letter *T*) and experimental (denoted by the letter *E*) values of the parameters $\Phi$, $\Theta$, $\alpha$, $\beta$, the output power, as well as the coefficients ($c_{1}$ and $c_{2}$) for two eigenstates, $\left| -1,L \right\rangle$ and $\left| +1,R \right\rangle$. Figures S3A.1-3 are the conventional characterizations of the intracavity controlled OAM-carrying femtosecond laser pulses.

**Figure S2.** Simulations of the mean HOP sphere ($\bar{\boldsymbol{S}}$) representation of vector vortex states corresponding to the center wavelength ($\lambda_{0}$) and five closed paths for the laser intracavity modulation on the HOP sphere. The left image of every pair of figures demonstrates the intensity pattern of the output beam cross-section, while the right image is the transmitted intensity from a linear polarizer oriented in the parallel direction, denoted by the white double-ended arrow. The red single-ended arrows show the distributions of the polarization ellipses. $\Phi$ and $\Theta$ are the polar and azimuthal angles of the spherical coordinates, respectively. ${\bar{\boldsymbol{S}}}_{\boldsymbol{i}}^{\boldsymbol{+1}} (i=1,2,3)$ denotes the three axes of the HOP sphere where +1 is the topological charge corresponding to $2q=+1$. Red circle: Path A, the equator; green circle: Path B, the circle of 0° and 180° longitude; blue circle: Path D, the circle of 45° latitude; cyan circle: Path E, the circle of −45° latitude.


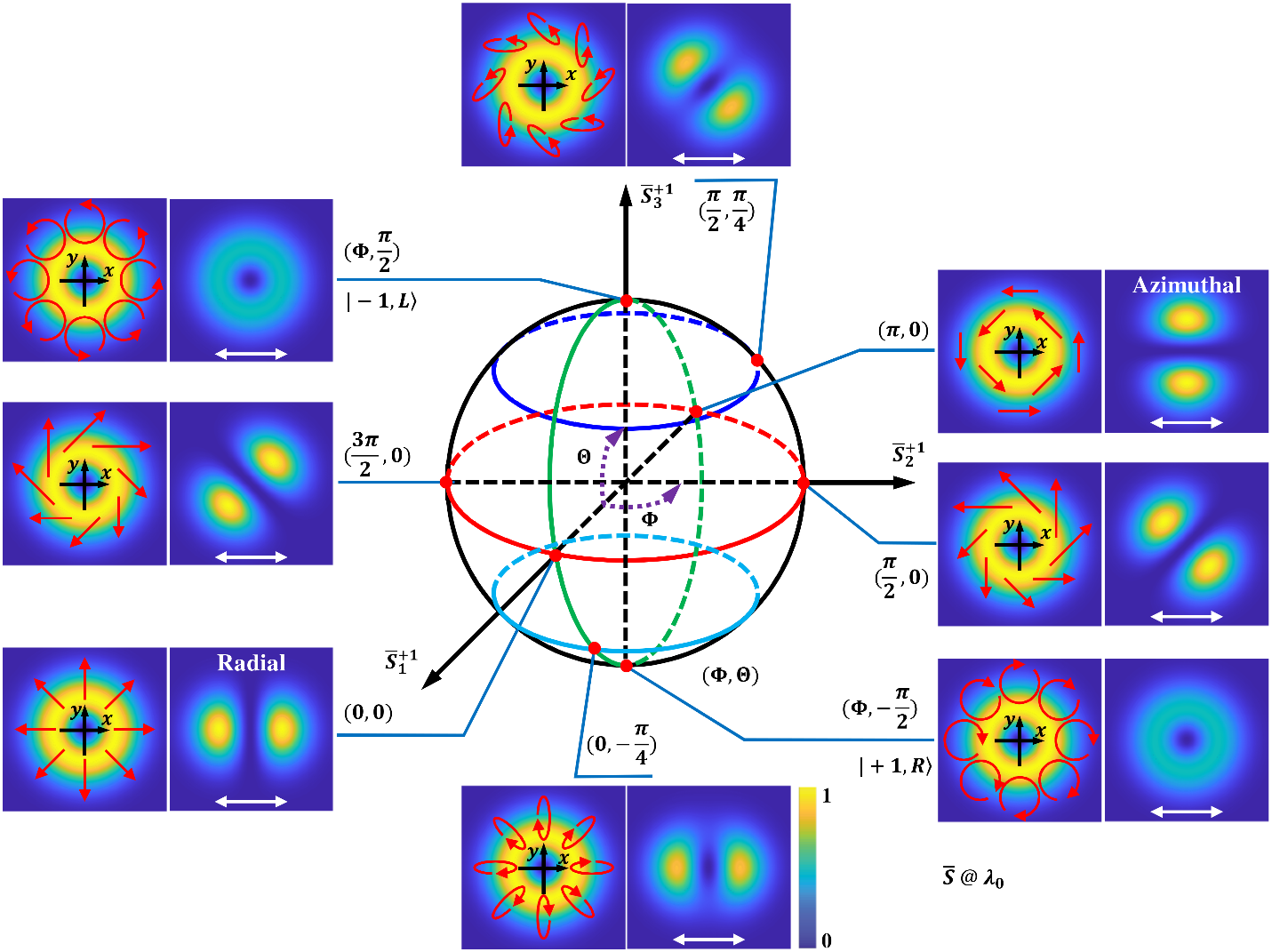


| **Index** | $\boldsymbol{\Phi}_{\mathbf{T}}$  **(rad.)** | $\boldsymbol{\Theta}_{\mathbf{T}}$  **(rad.)** | $\boldsymbol{\alpha}_{\mathbf{T}}$  **(rad.)** | $\boldsymbol{\beta}_{\mathbf{T}}$  **(rad.)** | $\boldsymbol{\alpha}_{\mathbf{T}}$  **(deg.)** | $\boldsymbol{\beta}_{\mathbf{T}}$  **(deg.)** | **Power (mW)** | $\boldsymbol{c}_{\boldsymbol{1}}$ | $\boldsymbol{c}_{\boldsymbol{2}}$ |
| --- | --- | --- | --- | --- | --- | --- | --- | --- | --- |
| **1** | 0 | 0 | 0 | 0 | 352.0 | 355.1 | 3.493 | $\frac{1}{\sqrt{2}}$ | $\frac{1}{\sqrt{2}}$ |
| **2** | $\frac{\pi}{4}$ | 0 | $\frac{\pi}{8}$ | 0 | 14.5 | 355.1 | 3.301 | $\frac{1}{\sqrt{2}}exp(-\frac{\pi}{8}i)$ | $\frac{1}{\sqrt{2}}exp(\frac{\pi}{8}i)$ |
| **3** | $\frac{\pi}{2}$ | 0 | $\frac{\pi}{4}$ | 0 | 37.0 | 355.1 | 3.173 | $\frac{1}{\sqrt{2}}exp(-\frac{\pi}{4}i)$ | $\frac{1}{\sqrt{2}}exp(\frac{\pi}{4}i)$ |
| **4** | $\frac{3\pi}{4}$ | 0 | $\frac{3\pi}{8}$ | 0 | 59.5 | 355.1 | 3.191 | $\frac{1}{\sqrt{2}}exp(-\frac{3\pi}{8}i)$ | $\frac{1}{\sqrt{2}}exp(\frac{3\pi}{8}i)$ |
| **5** | $\pi$ | 0 | $\frac{\pi}{2}$ | 0 | 82.0 | 355.1 | 3.338 | $-\frac{1}{\sqrt{2}}i$ | $\frac{1}{\sqrt{2}}i$ |
| **6** | $\frac{5\pi}{4}$ | 0 | $\frac{5\pi}{8}$ | 0 | 104.5 | 355.1 | 3.490 | $\frac{1}{\sqrt{2}}exp(-\frac{5\pi}{8}i)$ | $\frac{1}{\sqrt{2}}exp(\frac{5\pi}{8}i)$ |
| **7** | $\frac{3\pi}{2}$ | 0 | $\frac{3\pi}{4}$ | 0 | 127.0 | 355.1 | 3.442 | $\frac{1}{\sqrt{2}}exp(-\frac{3\pi}{4}i)$ | $\frac{1}{\sqrt{2}}exp(\frac{3\pi}{4}i)$ |
| **8** | $\frac{7\pi}{4}$ | 0 | $\frac{7\pi}{8}$ | 0 | 149.5 | 355.1 | 3.347 | $\frac{1}{\sqrt{2}}exp(-\frac{7\pi}{8}i)$ | $\frac{1}{\sqrt{2}}exp(\frac{7\pi}{8}i)$ |
| **9** | $2\pi$ | 0 | $\pi$ | 0 | 172.0 | 355.1 | 3.203 | $-\frac{1}{\sqrt{2}}$ | $-\frac{1}{\sqrt{2}}$ |

**Table 2SA.** List of the theoretical (*T*) and experimental (*E*) values of the parameters and coefficients of $\left| -1,L \right\rangle$ and $\left| +1,R \right\rangle$ modulated along Path A.
