# Supplementary material for: Integrated pulse scope for tunable generation and intrinsic characterization of structured femtosecond laser
Source: Sci Rep. 2021 May 6;11:9670. doi: 10.1038/s41598-021-87938-w (PMC8102529; doi:10.1038/s41598-021-87938-w)
Supplement: Supplementary file 2 — Supplementary Information 2. [file 41598_2021_87938_MOESM2_ESM.docx]

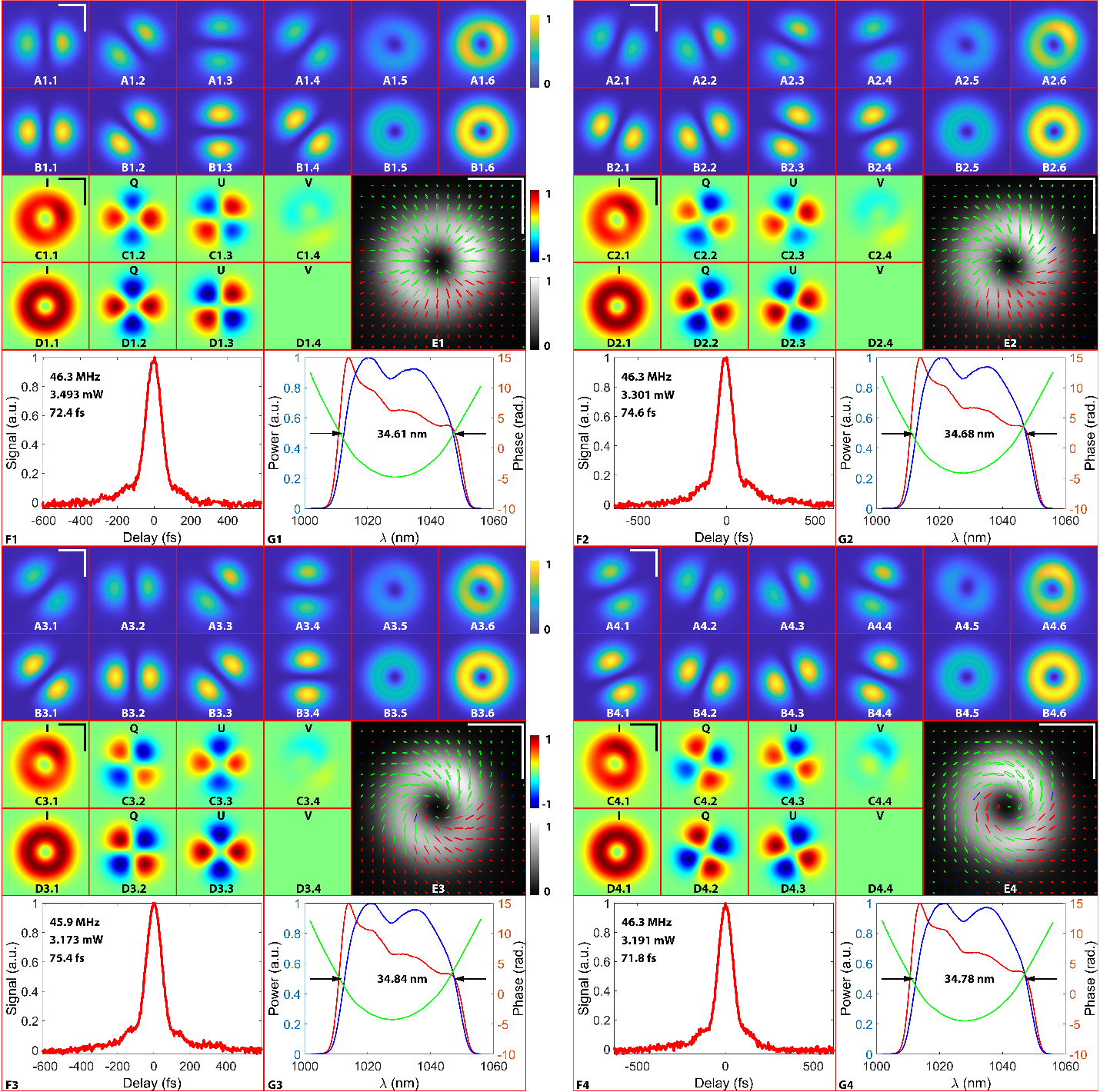


**Figure S3A1.** Conventional characterizations of the intracavity controlled OAM-carrying femtosecond laser with the states described by the $\left( 0\boldsymbol{,}0 \right)$, $\left( \pi/4,0 \right)$, $\left( \pi/2,0 \right)$ and $\left( 3\pi/4,0 \right)$ on the HOP sphere ($\overline{S}$). **A1-G1**: $(\left| -1,L \right\rangle+\left| +1,R \right\rangle)/\sqrt{2}$ corresponding to the radial state $\left( 0\boldsymbol{,}0 \right)$. **A2-G2**: $[exp\left( -\frac{\pi}{8}i \right)\left| -1,L \right\rangle+exp\left( \frac{\pi}{8}i \right)\left| +1,R \right\rangle]/\sqrt{2}$ corresponding to the state $\left( \frac{\pi}{4},0 \right)$. **A3-G3**: $[exp\left( -\frac{\pi}{4}i \right)\left| -1,L \right\rangle+exp\left( \frac{\pi}{4}i \right)\left| +1,R \right\rangle]/\sqrt{2}$ corresponding to the point $\left( \frac{\pi}{2},0 \right)$. **A4-G4**:$[exp\left( -\frac{3\pi}{8}i \right)\left| -1,L \right\rangle+exp\left( \frac{3\pi}{8}i \right)\left| +1,R \right\rangle]/\sqrt{2}$ corresponding to the point $\left( \frac{3\pi}{4}\boldsymbol{,}0 \right)$. For the radial state: **A1.1**-**A1.6** are the 2D intensity measurements with different configurations. **A1.6** are the total intensity distributions; **A1.5** are the patterns after the pulses transmit through the QWP with horizontal fast axis and the polarizer with the orientation of $45^{\circ}$; **A1.1**-**A1.4** are the patterns when the polarizers with such orientations as: $1\to0^{\circ}$, $2\to45^{\circ}$, $3\to90^{\circ}$,$4\to135^{\circ}$; **C1.1**-**C1.4** are the Stokes parameters; **B1.1**-**B1.6**, and **D1.1**-**D1.4** are the corresponding simulations; **E1** are the polarization ellipses, where green denotes the left-handed polarization, red the right-handed polarization, and blue the linearly polarized; **F1** are the pulse duration measurements of the pulse from the inner cavity (reference beam)after the chirp compensation (The inserted numbers are, repetition rate, average output power of the sample pulse, and the pulse duration of the reference pulse with the assumption that the dechirped pulse carries with the Gaussian shape, respectively.); **G1** is the spectrum of the pulses from the laser output coupler (blue), the reference beam (red) as well as the phase (green) (The inserted numbers is the FWHM of the blue curve.). The right portion of this figure shares the same setup. Scale bars represent 1 mm.

**Figure S3A2.** Conventional characterizations of the states described by the $\left( \pi\boldsymbol{,}0 \right)$, $\left( 5\pi/4,0 \right)$, $\left( 3\pi/2,0 \right)$, and $\left( 7\pi/4,0 \right)$ on $\overline{S}$. **A1-G1**: $(-\left| -1,L \right\rangle+\left| +1,R \right\rangle)i/\sqrt{2}$ corresponding to the radial state $\left( \pi\boldsymbol{,}0 \right)$. **A2-G2**: $[exp\left( -\frac{5\pi}{8}i \right)\left| -1,L \right\rangle+exp\left( \frac{5\pi}{8}i \right)\left| +1,R \right\rangle]/\sqrt{2}$ corresponding to the state $\left( \frac{5\pi}{4},0 \right)$. **A3-G3**: $[exp\left( -\frac{3\pi}{4}i \right)\left| -1,L \right\rangle+exp\left( \frac{3\pi}{4}i \right)\left| +1,R \right\rangle]/\sqrt{2}$ corresponding to the point $\left( \frac{3\pi}{2},0 \right)$. **A4-G4**:$[exp\left( -\frac{7\pi}{8}i \right)\left| -1,L \right\rangle+exp\left( \frac{7\pi}{8}i \right)\left| +1,R \right\rangle]/\sqrt{2}$ corresponding to the point $\left( \frac{7\pi}{4}\boldsymbol{,}0 \right)$.


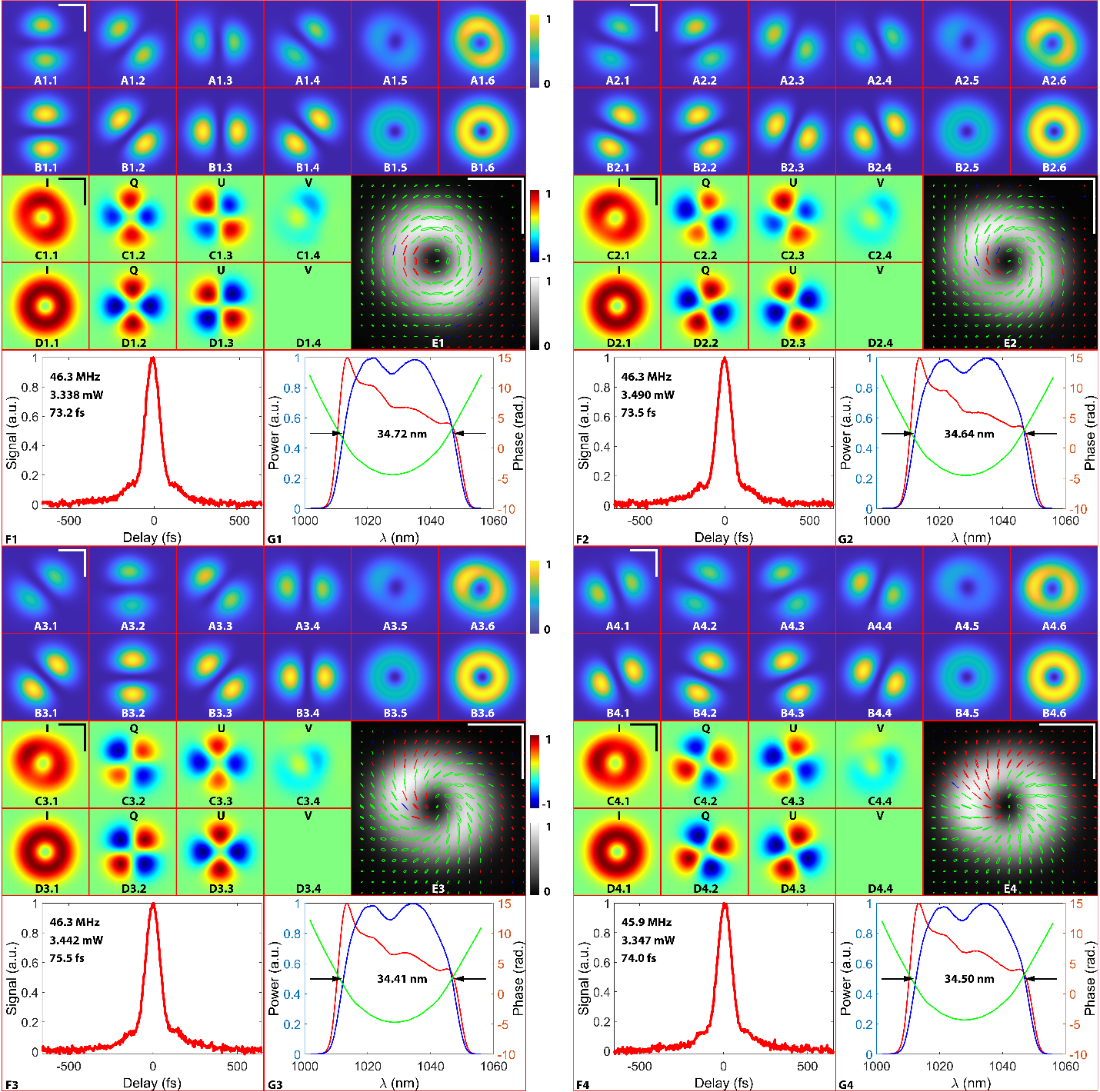


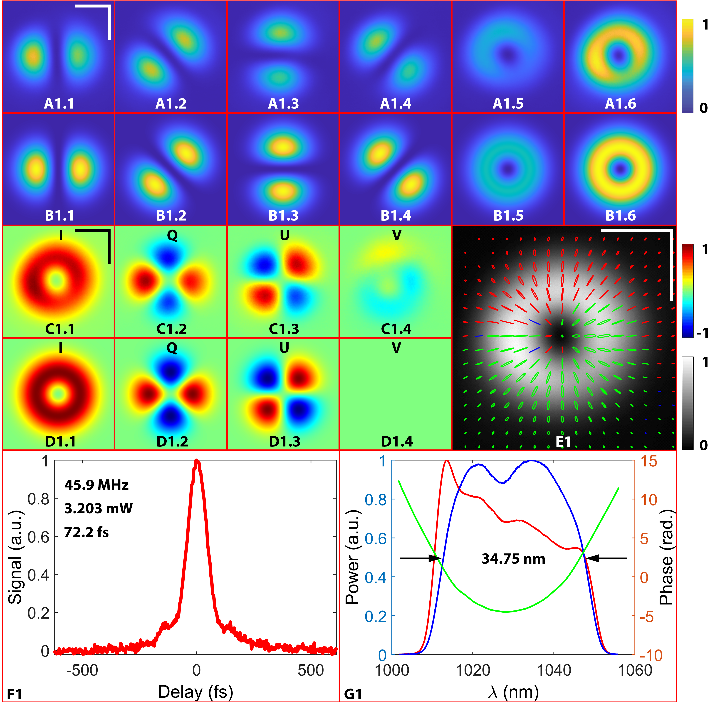


**Figure S3A3.** Conventional characterization of the states described by the $\left( 2\pi\boldsymbol{,}0 \right)$ on $\overline{S}$. **A1-G1**: $(\left| -1,L \right\rangle+\left| +1,R \right\rangle)/\sqrt{2}$ corresponding to the radial state $\left( 2\pi\boldsymbol{,}0 \right)$.

**I.3.B. Path B, the Circle of 0° and 180° Longitude**

This section presents the detailed characterization for the states modulated along the Path B. Table S2B shows the *T* and *E* values of $\Phi$, $\Theta$, $\alpha$, $\beta$, the output power, $c_{1}$ and $c_{2}$ for two eigenstates, $\left| -1,L \right\rangle$ and $\left| +1,R \right\rangle$. Figures S3B.1-3 are the conventional characterizations.

| **Index** | $\boldsymbol{\Phi}_{\mathbf{T}}$  **(rad.)** | $\boldsymbol{\Theta}_{\mathbf{T}}$  **(rad.)** | $\boldsymbol{\alpha}_{\mathbf{T}}$  **(rad.)** | $\boldsymbol{\beta}_{\mathbf{T}}$  **(rad.)** | $\boldsymbol{\alpha}_{\mathbf{T}}$  **(deg.)** | $\boldsymbol{\beta}_{\mathbf{T}}$  **(deg.)** | **Power (mW)** | $\boldsymbol{c}_{\boldsymbol{1}}$ | $\boldsymbol{c}_{\boldsymbol{2}}$ |
| --- | --- | --- | --- | --- | --- | --- | --- | --- | --- |
| **1** | 0 | 0 | 0 | 0 | 352 | 355.1 | 3.656 | $\frac{1}{\sqrt{2}}$ | $\frac{1}{\sqrt{2}}$ |
| **2** | $0$ | $\frac{\pi}{4}$ | $\frac{\pi}{8}$ | $\frac{\pi}{8}$ | 14.5 | 332.6 | 3.348 | $sin(\frac{3\pi}{8})$ | $cos(\frac{3\pi}{8})$ |
| **3** | 0 | $\frac{\pi}{2}$ | $\frac{\pi}{4}$ | $\frac{\pi}{4}$ | 37 | 310.1 | 3.444 | $1$ | $0$ |
| **4** | $\pi$ | $\frac{\pi}{4}$ | $\frac{5\pi}{8}$ | $\frac{\pi}{8}$ | 104.5 | 332.6 | 3.474 | $-sin(\frac{3\pi}{8})i$ | $cos(\frac{3\pi}{8})i$ |
| **5** | $\pi$ | 0 | $\frac{\pi}{2}$ | 0 | 82 | 355.1 | 3.225 | $-\frac{1}{\sqrt{2}}i$ | $\frac{1}{\sqrt{2}}i$ |
| **6** | $\pi$ | $-\frac{\pi}{4}$ | $\frac{3\pi}{8}$ | $-\frac{\pi}{8}$ | 59.5 | 17.6 | 3.182 | $-sin(\frac{\pi}{8})i$ | $cos(\frac{\pi}{8})i$ |
| **7** | $\pi$ | $-\frac{\pi}{2}$ | $\frac{\pi}{4}$ | $-\frac{\pi}{4}$ | 37 | 40.1 | 3.381 | $0$ | $i$ |
| **8** | $0$ | $-\frac{\pi}{4}$ | $-\frac{\pi}{8}$ | $-\frac{\pi}{8}$ | 329.5 | 17.6 | 3.661 | $sin(\frac{\pi}{8})$ | $cos(\frac{\pi}{8})$ |
| **9** | $0$ | 0 | $0$ | 0 | 352 | 355.1 | 3.477 | $\frac{1}{\sqrt{2}}$ | $\frac{1}{\sqrt{2}}$ |

**Table S2B.** List of the *T* and *E* values of the parameters and coefficients of $\left| -1,L \right\rangle$ and $\left| +1,R \right\rangle$ modulated along Path B.


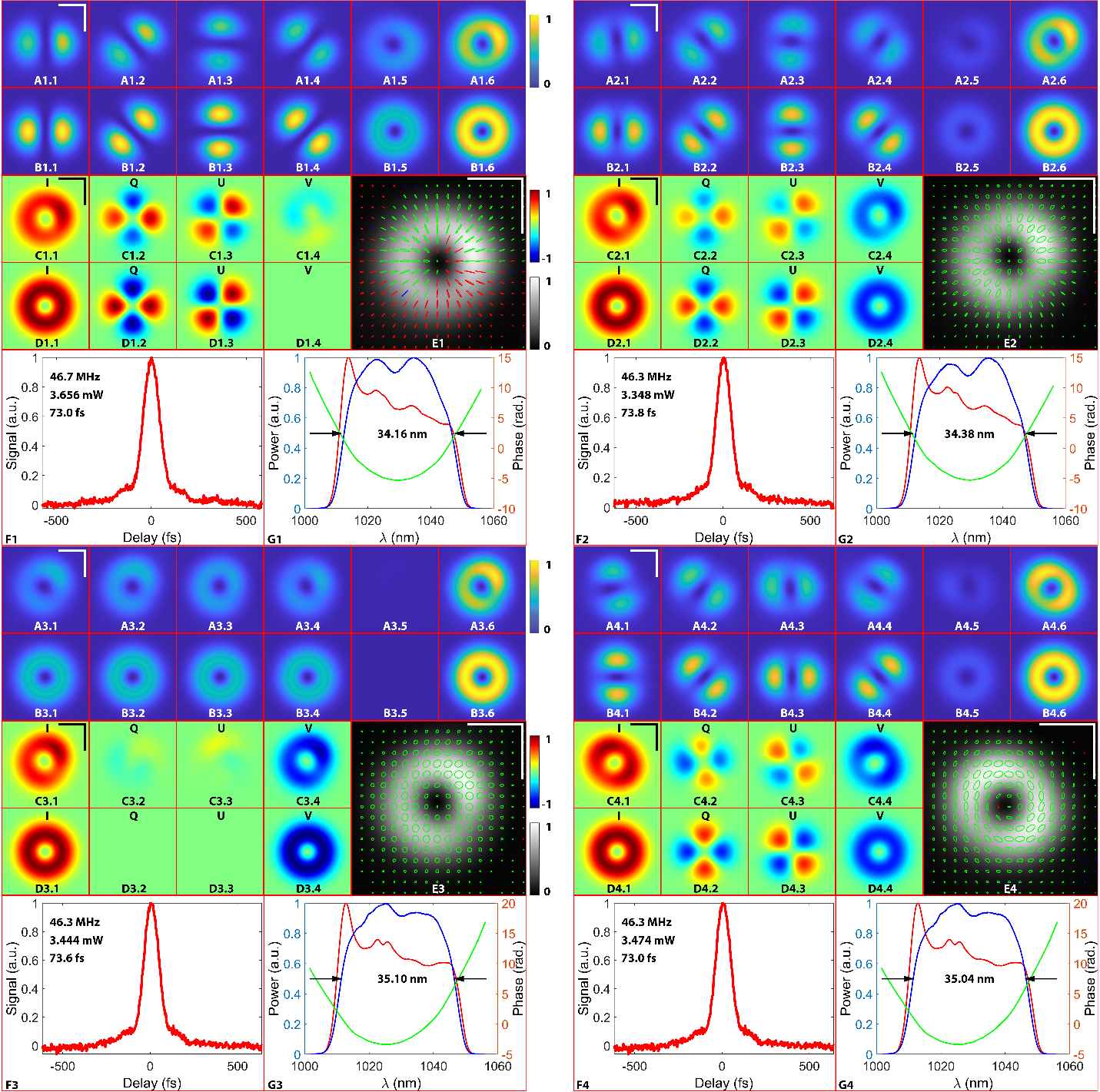


**Figure S3B1.** Conventional characterizations of the states described by $\left( 0\boldsymbol{,}0 \right)$, $\left( 0,\pi/4 \right)$, $\left( 0,\pi/2 \right)$, and $\left( \pi,\pi/4 \right)$ on $\overline{S}$. **A1-G1**: $(\left| -1,L \right\rangle+\left| +1,R \right\rangle)/\sqrt{2}$ corresponding to the radial state $\left( 0\boldsymbol{,}0 \right)$. **A2-G2**: $[sin(\frac{3\pi}{8})\left| -1,L \right\rangle+cos(\frac{3\pi}{8})\left| +1,R \right\rangle]/\sqrt{2}$ corresponding to the state $\left( 0,\frac{\pi}{4} \right)$. **A3-G3**: $[\left| -1,L \right\rangle+0\left| +1,R \right\rangle]/\sqrt{2}$ corresponding to the point $\left( 0,\frac{\pi}{2} \right)$. **A4-G4**:$[-sin(\frac{3\pi}{8})\left| -1,L \right\rangle+cos(\frac{3\pi}{8})\left| +1,R \right\rangle]i/\sqrt{2}$ corresponding to the point $\left( \pi,\pi/4 \right)$.


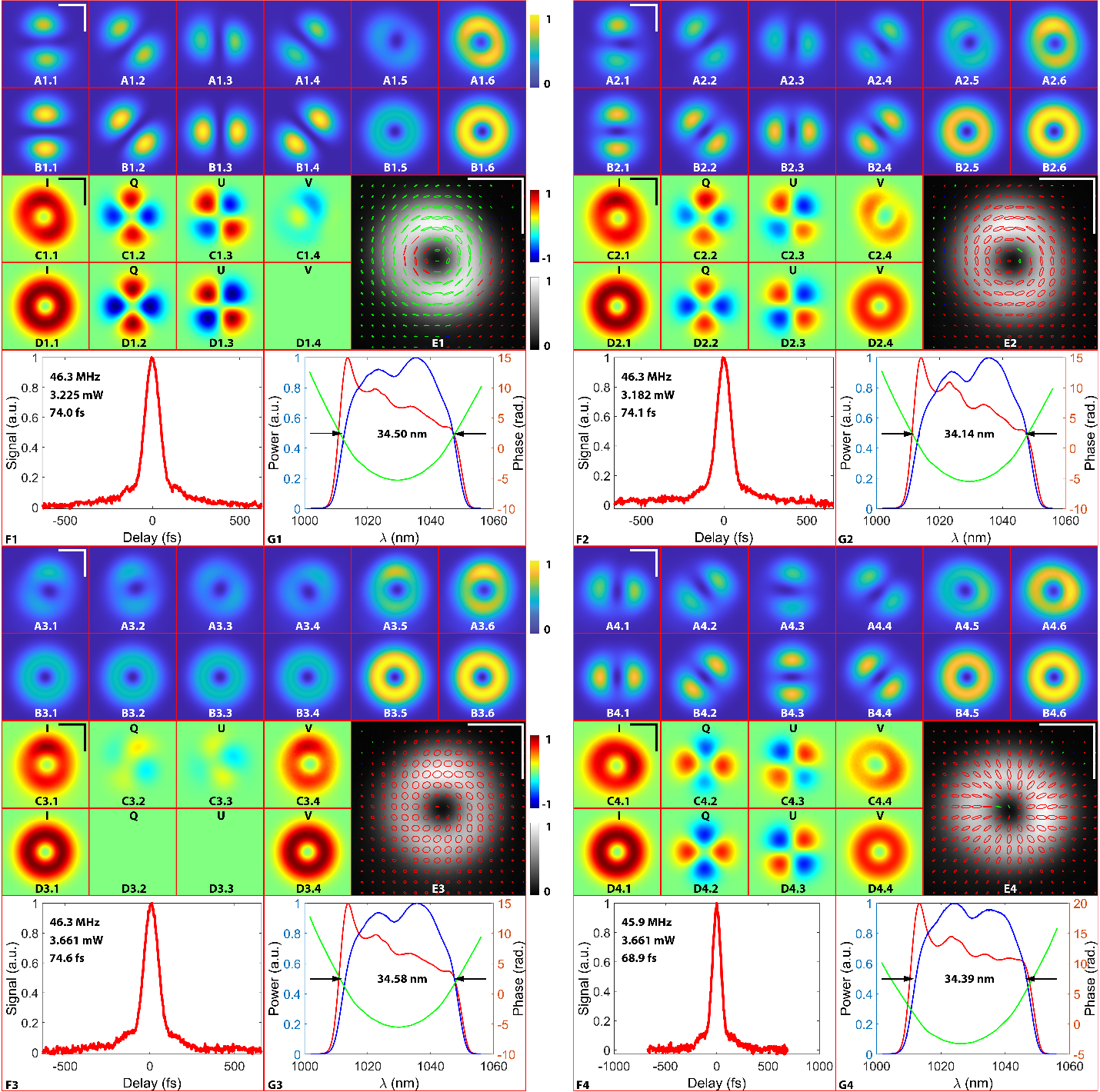


**Figure S3B2.** Conventional characterizations of the states described by $\left( \pi\boldsymbol{,}0 \right)$, $\left( \pi,-\pi/4 \right)$, $\left( \pi,-\pi/2 \right)$, and $\left( 0,-\pi/4 \right)$ on $\overline{S}$. **A1-G1**: $(-\left| -1,L \right\rangle+\left| +1,R \right\rangle)i/\sqrt{2}$ corresponding to the radial state $\left( \pi\boldsymbol{,}0 \right)$. **A2-G2**: $[-sin(\frac{\pi}{8})\left| -1,L \right\rangle+cos(\frac{\pi}{8})\left| +1,R \right\rangle]i/\sqrt{2}$ corresponding to the state $\left( \pi,-\pi/4 \right)$. **A3-G3**: $[0\left| -1,L \right\rangle+i\left| +1,R \right\rangle]/\sqrt{2}$corresponding to the point $\left( \pi,-\pi/2 \right)$. **A4-G4**:$[sin(\frac{\pi}{8})\left| -1,L \right\rangle+cos(\frac{\pi}{8})\left| +1,R \right\rangle]/\sqrt{2}$ corresponding to the point $\left( 0,-\pi/4 \right)$.


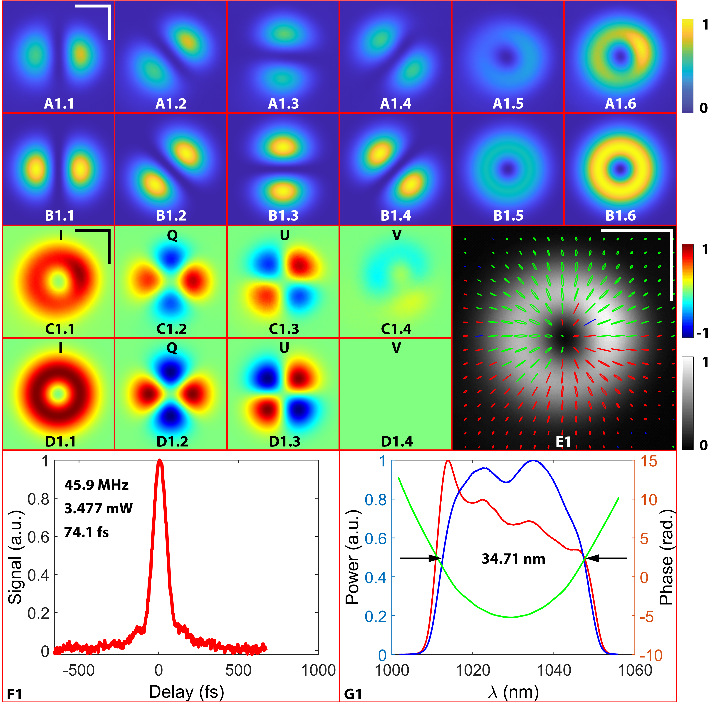


**Figure S3B3.** Conventional characterization of the state $\left( 0\boldsymbol{,}0 \right)$ on $\overline{S}$. **A1-G1**: $(\left| -1,L \right\rangle+\left| +1,R \right\rangle)/\sqrt{2}$ corresponding to the radial state $\left( 0\boldsymbol{,}0 \right)$.

**I.3.C. Path C, the Polar Triangle**

This part shows the characterizations of the states modulated along Path C. Table S2C shows the *T* and *E* values. Figures 3C.1-3 are the conventional characterizations.

| **Index** | $\boldsymbol{\Phi}_{\mathbf{T}}$  **(rad.)** | $\boldsymbol{\Theta}_{\mathbf{T}}$  **(rad.)** | $\boldsymbol{\alpha}_{\mathbf{T}}$  **(rad.)** | $\boldsymbol{\beta}_{\mathbf{T}}$  **(rad.)** | $\boldsymbol{\alpha}_{\mathbf{T}}$  **(deg.)** | $\boldsymbol{\beta}_{\mathbf{T}}$  **(deg.)** | **Power (mW)** | $\boldsymbol{c}_{\boldsymbol{1}}$ | $\boldsymbol{c}_{\boldsymbol{2}}$ |
| --- | --- | --- | --- | --- | --- | --- | --- | --- | --- |
| **1** | 0 | 0 | 0 | 0 | 352 | 355.1 | 3.413 | $\frac{1}{\sqrt{2}}$ | $\frac{1}{\sqrt{2}}$ |
| **2** | $\frac{\pi}{4}$ | 0 | $\frac{\pi}{8}$ | 0 | 14.5 | 355.1 | 3.194 | $\frac{1}{\sqrt{2}}exp(-\frac{\pi}{8}i)$ | $\frac{1}{\sqrt{2}}exp(\frac{\pi}{8}i)$ |
| **3** | $\frac{\pi}{2}$ | 0 | $\frac{\pi}{4}$ | 0 | 37 | 355.1 | 3.061 | $\frac{1}{\sqrt{2}}exp(-\frac{\pi}{4}i)$ | $\frac{1}{\sqrt{2}}exp(\frac{\pi}{4}i)$ |
| **4** | $\frac{\pi}{2}$ | $\frac{\pi}{4}$ | $\frac{3\pi}{8}$ | $\frac{\pi}{8}$ | 59.5 | 332.6 | 3.294 | $sin(\frac{3\pi}{8})exp(-\frac{\pi}{4}i)$ | $cos(\frac{3\pi}{8})exp(\frac{\pi}{4}i)$ |
| **5** | $\frac{\pi}{2}$ | $\frac{\pi}{2}$ | $\frac{\pi}{2}$ | $\frac{\pi}{4}$ | 82 | 310.1 | 3.675 | $exp(-\frac{\pi}{4}i)$ | $0$ |
| **6** | 0 | $\frac{\pi}{2}$ | $\frac{\pi}{4}$ | $\frac{\pi}{4}$ | 37 | 310.1 | 3.257 | $1$ | $0$ |
| **7** | $0$ | $\frac{\pi}{4}$ | $\frac{\pi}{8}$ | $\frac{\pi}{8}$ | 14.5 | 332.6 | 3.182 | $sin(\frac{3\pi}{8})$ | $cos(\frac{3\pi}{8})$ |
| **8** | $0$ | 0 | $0$ | 0 | 352 | 355.1 | 3.413 | $\frac{1}{\sqrt{2}}$ | $\frac{1}{\sqrt{2}}$ |

**Table S2C.** List of *T* and *E* values for $\left| -1,L \right\rangle$ and $\left| +1,R \right\rangle$ modulated along Path C.
