# Supplementary material for: Integrated pulse scope for tunable generation and intrinsic characterization of structured femtosecond laser
Source: Sci Rep. 2021 May 6;11:9670. doi: 10.1038/s41598-021-87938-w (PMC8102529; doi:10.1038/s41598-021-87938-w)
Supplement: Supplementary file 3 — Supplementary Information 3. [file 41598_2021_87938_MOESM3_ESM.docx]

**Figure S3C1.** Conventional characterizations of the states $\left( 0\boldsymbol{,}0 \right)$, $\left( \pi/4,0 \right)$, $\left( \pi/2,0 \right)$, and $\left( \pi/2,\pi/4 \right)$ on $\overline{S}$. **A1-G1**: $(\left| -1,L \right\rangle+\left| +1,R \right\rangle)/\sqrt{2}$ corresponding to the radial state $\left( 0\boldsymbol{,}0 \right)$. **A2-G2**: $[exp\left( -\frac{\pi}{8}i \right)\left| -1,L \right\rangle+exp\left( \frac{\pi}{8}i \right)\left| +1,R \right\rangle]/\sqrt{2}$ corresponding to the state $\left( \frac{\pi}{4},0 \right)$. **A3-G3**: $[exp\left( -\frac{\pi}{4}i \right)\left| -1,L \right\rangle+exp\left( \frac{\pi}{4}i \right)\left| +1,R \right\rangle]/\sqrt{2}$corresponding to the point $\left( \frac{\pi}{2},0 \right)$. **A4-G4**:$[sin(\frac{3\pi}{8})exp(-\frac{\pi}{4}i)\left| -1,L \right\rangle+cos(\frac{3\pi}{8})exp(\frac{\pi}{4}i)\left| +1,R \right\rangle]/\sqrt{2}$ corresponding to the point $\left( \frac{\pi}{2},\frac{\pi}{4} \right)$.


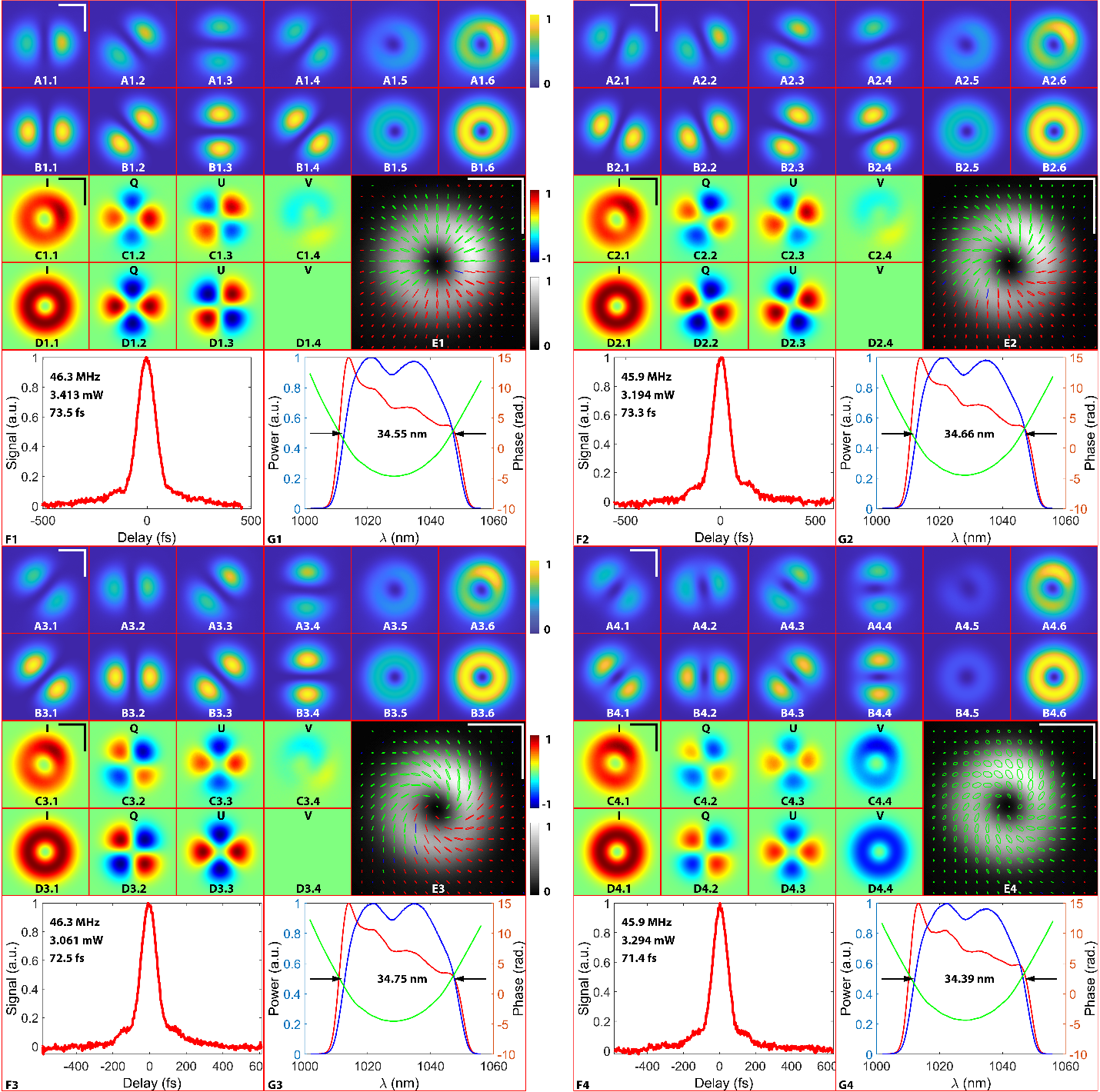


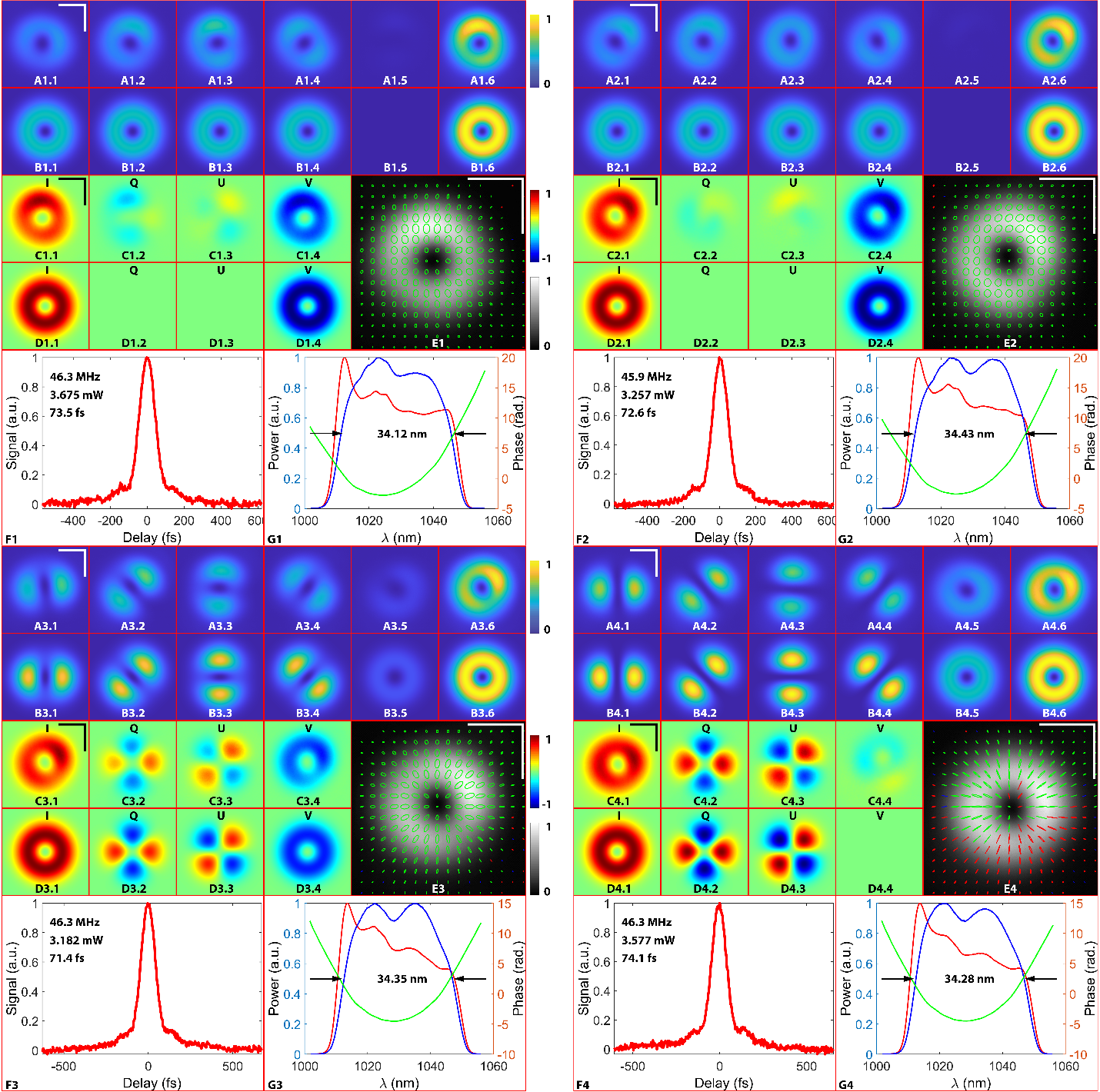


**Figure S3C2.** Conventional characterizations of the states described by the $\left( \pi/2\boldsymbol{,}\pi/2 \right)$, $\left( 0, \pi/2 \right)$, $\left( 0, \pi/4 \right)$, and $\left( 0,0 \right)$ on $\overline{S}$. **A1-G1**: $[exp(-\frac{\pi}{4}i)\left| -1,L \right\rangle+0\left| +1,R \right\rangle]/\sqrt{2}$ corresponding to the radial state $\left( \pi/2\boldsymbol{,}\pi/2 \right)$. **A2-G2**: $(1\left| -1,L \right\rangle+0\left| +1,R \right\rangle)/\sqrt{2}$ corresponding to the state $\left( 0,\frac{\pi}{2} \right)$. **A3-G3**: $[sin(\frac{3\pi}{8})\left| -1,L \right\rangle+cos(\frac{3\pi}{8})\left| +1,R \right\rangle]/\sqrt{2}$corresponding to the point $\left( 0,\frac{\pi}{4} \right)$. **A4-G4**:$(\left| -1,L \right\rangle+\left| +1,R \right\rangle)/\sqrt{2}$ corresponding to the point $\left( 0,0 \right)$.

**I.3.D. Path D, the Circle of 45° Latitude**

This part shows the characterizations of the states modulated along the path D. Table S2D shows the *T* and *E* values. Figures 3D.1-3 are the conventional characterizations.

| **Index** | $\boldsymbol{\Phi}_{\mathbf{T}}$  **(rad.)** | $\boldsymbol{\Theta}_{\mathbf{T}}$  **(rad.)** | $\boldsymbol{\alpha}_{\mathbf{T}}$  **(rad.)** | $\boldsymbol{\beta}_{\mathbf{T}}$  **(rad.)** | $\boldsymbol{\alpha}_{\mathbf{T}}$  **(deg.)** | $\boldsymbol{\beta}_{\mathbf{T}}$  **(deg.)** | **Power (mW)** | $\boldsymbol{c}_{\boldsymbol{1}}$ | $\boldsymbol{c}_{\boldsymbol{2}}$ |
| --- | --- | --- | --- | --- | --- | --- | --- | --- | --- |
| **1** | 0 | $\frac{\pi}{4}$ | $\frac{\pi}{8}$ | $\frac{\pi}{8}$ | 14.5 | 332.6 | 3.319 | $sin(\frac{3\pi}{8})$ | $cos(\frac{3\pi}{8})$ |
| **2** | $\frac{\pi}{4}$ | $\frac{\pi}{4}$ | $\frac{\pi}{4}$ | $\frac{\pi}{8}$ | 37 | 332.6 | 3.194 | $sin(\frac{3\pi}{8})exp(-\frac{\pi}{8}i)$ | $cos(\frac{3\pi}{8})exp(\frac{\pi}{8}i)$ |
| **3** | $\frac{\pi}{2}$ | $\frac{\pi}{4}$ | $\frac{3\pi}{8}$ | $\frac{\pi}{8}$ | 59.5 | 332.6 | 3.22 | $sin(\frac{3\pi}{8})exp(-\frac{\pi}{4}i)$ | $cos(\frac{3\pi}{8})exp(\frac{\pi}{4}i)$ |
| **4** | $\frac{3\pi}{4}$ | $\frac{\pi}{4}$ | $\frac{\pi}{2}$ | $\frac{\pi}{8}$ | 82 | 332.6 | 3.338 | $sin(\frac{3\pi}{8})exp(-\frac{3\pi}{8}i)$ | $cos(\frac{3\pi}{8})exp(\frac{3\pi}{8}i)$ |
| **5** | $\pi$ | $\frac{\pi}{4}$ | $\frac{5\pi}{8}$ | $\frac{\pi}{8}$ | 104.5 | 332.6 | 3.492 | $-sin(\frac{3\pi}{8})i$ | $cos(\frac{3\pi}{8})i$ |
| **6** | $\frac{5\pi}{4}$ | $\frac{\pi}{4}$ | $\frac{3\pi}{4}$ | $\frac{\pi}{8}$ | 127 | 332.6 | 3.592 | $sin(\frac{3\pi}{8})exp(-\frac{5\pi}{8}i)$ | $cos(\frac{3\pi}{8})exp(\frac{5\pi}{8}i)$ |
| **7** | $\frac{3\pi}{2}$ | $\frac{\pi}{4}$ | $\frac{7\pi}{8}$ | $\frac{\pi}{8}$ | 149.5 | 332.6 | 3.542 | $sin(\frac{3\pi}{8})exp(-\frac{3\pi}{4}i)$ | $cos(\frac{3\pi}{8})exp(\frac{3\pi}{4}i)$ |
| **8** | $\frac{7\pi}{4}$ | $\frac{\pi}{4}$ | $\pi$ | $\frac{\pi}{8}$ | 172 | 332.6 | 3.446 | $sin(\frac{3\pi}{8})exp(-\frac{7\pi}{8}i)$ | $cos(\frac{3\pi}{8})exp(\frac{7\pi}{8}i)$ |
| **9** | $2\pi$ | $\frac{\pi}{4}$ | $\frac{9\pi}{8}$ | $\frac{\pi}{8}$ | 194.5 | 332.6 | 3.379 | $-sin(\frac{3\pi}{8})$ | $-cos(\frac{3\pi}{8})$ |
| **10** | 2$\pi$ | 0 | 0 | 0 | 352 | 355.1 | 3.51 | $-\frac{1}{\sqrt{2}}$ | $-\frac{1}{\sqrt{2}}$ |

**Table S2D.** List of *T* and *E* values for $\left| -1,L \right\rangle$ and $\left| +1,R \right\rangle$ modulated along Path D.

**Figure S3D1.** Conventional characterizations of the states $\left( 0\boldsymbol{,}\pi/4 \right)$, $\left( \pi/4,\pi/4 \right)$, $\left( \pi/2,\pi/4 \right)$, and $\left( 3\pi/4,\pi/4 \right)$ on $\overline{S}$. **A1-G1**: $(sin(\frac{3\pi}{8})\left| -1,L \right\rangle+cos(\frac{3\pi}{8})\left| +1,R \right\rangle)/\sqrt{2}$ corresponding to the radial state $\left( 0\boldsymbol{,}\pi/4 \right)$. **A2-G2**: $[sin(\frac{3\pi}{8})exp(-\frac{\pi}{8}i)\left| -1,L \right\rangle+cos(\frac{3\pi}{8})exp(\frac{\pi}{8}i)\left| +1,R \right\rangle]/\sqrt{2}$ corresponding to the state $\left( \pi/4,\pi/4 \right)$. **A3-G3**: $[sin(\frac{3\pi}{8})exp(-\frac{\pi}{4}i)\left| -1,L \right\rangle+cos(\frac{3\pi}{8})exp(\frac{\pi}{4}i)\left| +1,R \right\rangle]/\sqrt{2}$ corresponding to the point $\left( \pi/2,\pi/4 \right)$. **A4-G4**:$[sin(\frac{3\pi}{8})exp(-\frac{3\pi}{8}i)\left| -1,L \right\rangle+cos(\frac{3\pi}{8})exp(\frac{3\pi}{8}i)\left| +1,R \right\rangle]/\sqrt{2}$ corresponding to the point $\left( 3\pi/4,\pi/4 \right)$.


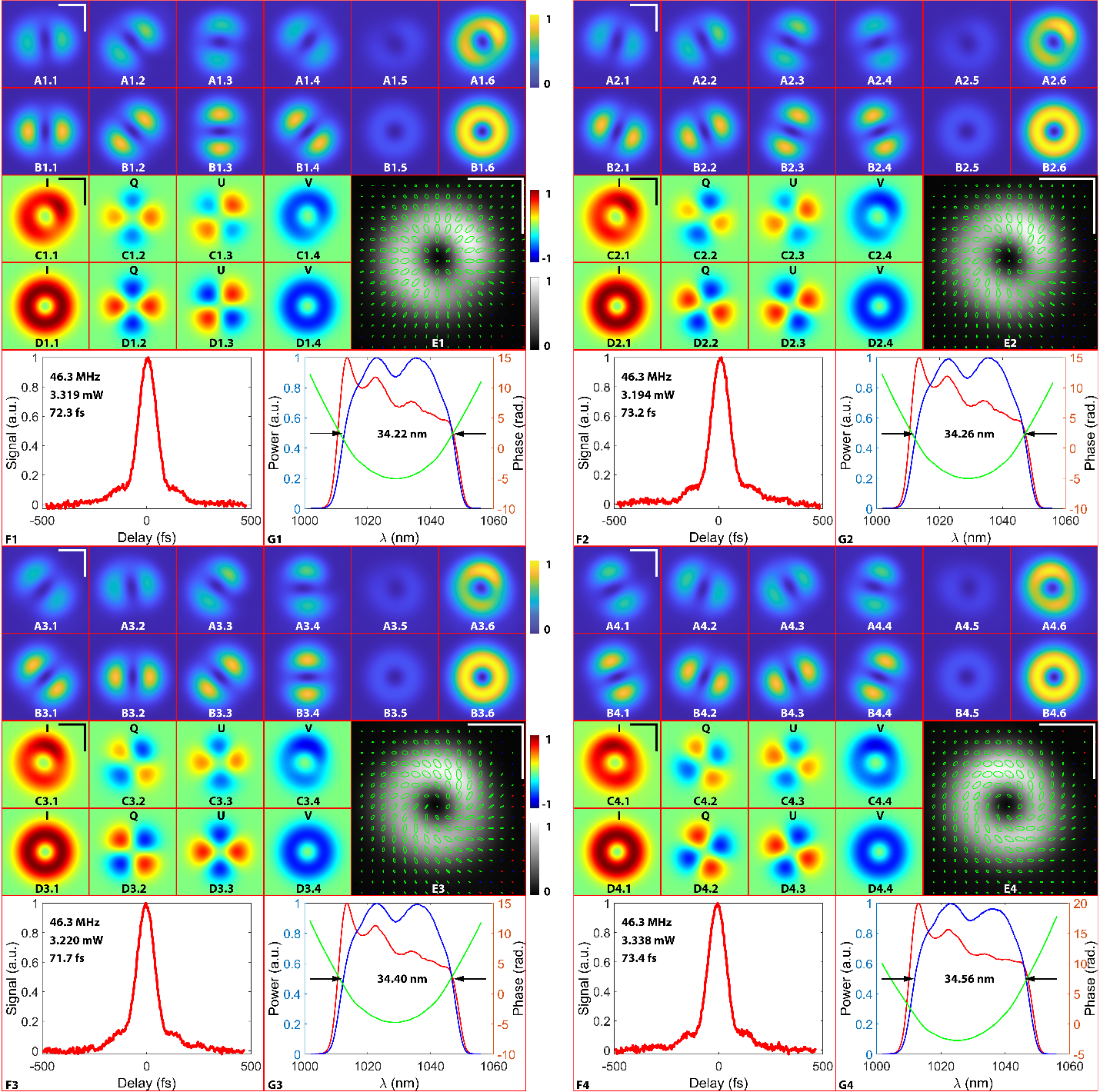


**Figure S3D2.** Conventional characterizations of the states $\left( \pi\boldsymbol{,}\pi/4 \right)$, $\left( 5\pi/4,\pi/4 \right)$, $\left( 3\pi/2,\pi/4 \right)$, and $\left( 7\pi/4,\pi/4 \right)$ on $\overline{S}$. **A1-G1**: $[-sin\left( \frac{3\pi}{8} \right)\left| -1,L \right\rangle+cos\left( \frac{3\pi}{8} \right)\left| +1,R \right\rangle]i/\sqrt{2}$ corresponding to the radial state $\left( \pi\boldsymbol{,}\pi/4 \right)$. **A2-G2**: $[sin(\frac{3\pi}{8})exp(-\frac{5\pi}{8}i)\left| -1,L \right\rangle+cos(\frac{3\pi}{8})exp(\frac{5\pi}{8}i)\left| +1,R \right\rangle]/\sqrt{2}$ corresponding to the state $\left( 5\pi/4,\pi/4 \right)$. **A3-G3**: $[sin(\frac{3\pi}{8})exp(-\frac{3\pi}{4}i)\left| -1,L \right\rangle+cos(\frac{3\pi}{8})exp(\frac{3\pi}{4}i)\left| +1,R \right\rangle]/\sqrt{2}$ corresponding to the point $\left( 3\pi/2,\pi/4 \right)$. **A4-G4**:$[sin(\frac{3\pi}{8})exp(-\frac{7\pi}{8}i)\left| -1,L \right\rangle+cos(\frac{3\pi}{8})exp(\frac{7\pi}{8}i)\left| +1,R \right\rangle]/\sqrt{2}$ corresponding to the point $\left( 7\pi/4,\pi/4 \right)$.


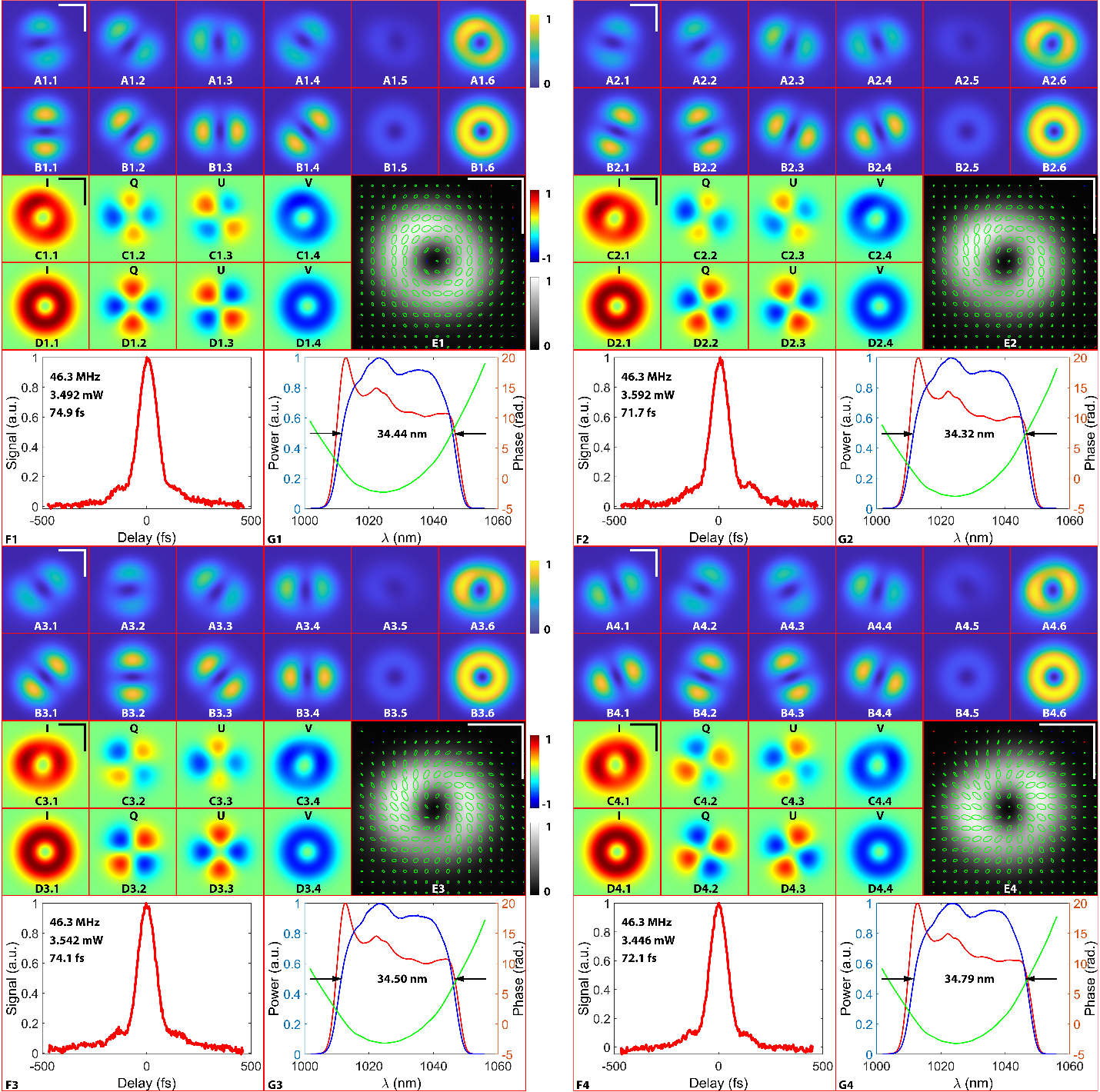


**Figure S3D3.** Conventional characterizations of the states $\left( 2\pi\boldsymbol{,}\pi/4 \right)$ and $\left( 2\pi,0 \right)$ on $\overline{S}$. **A1-G1**: $-[sin\left( \frac{3\pi}{8} \right)\left| -1,L \right\rangle+cos\left( \frac{3\pi}{8} \right)\left| +1,R \right\rangle]/\sqrt{2}$ corresponding to the radial state $\left( 2\pi\boldsymbol{,}\pi/4 \right)$. **A2-G2**: $-(\left| -1,L \right\rangle+\left| +1,R \right\rangle)/\sqrt{2}$ corresponding to the state $\left( 2\pi,0 \right)$.


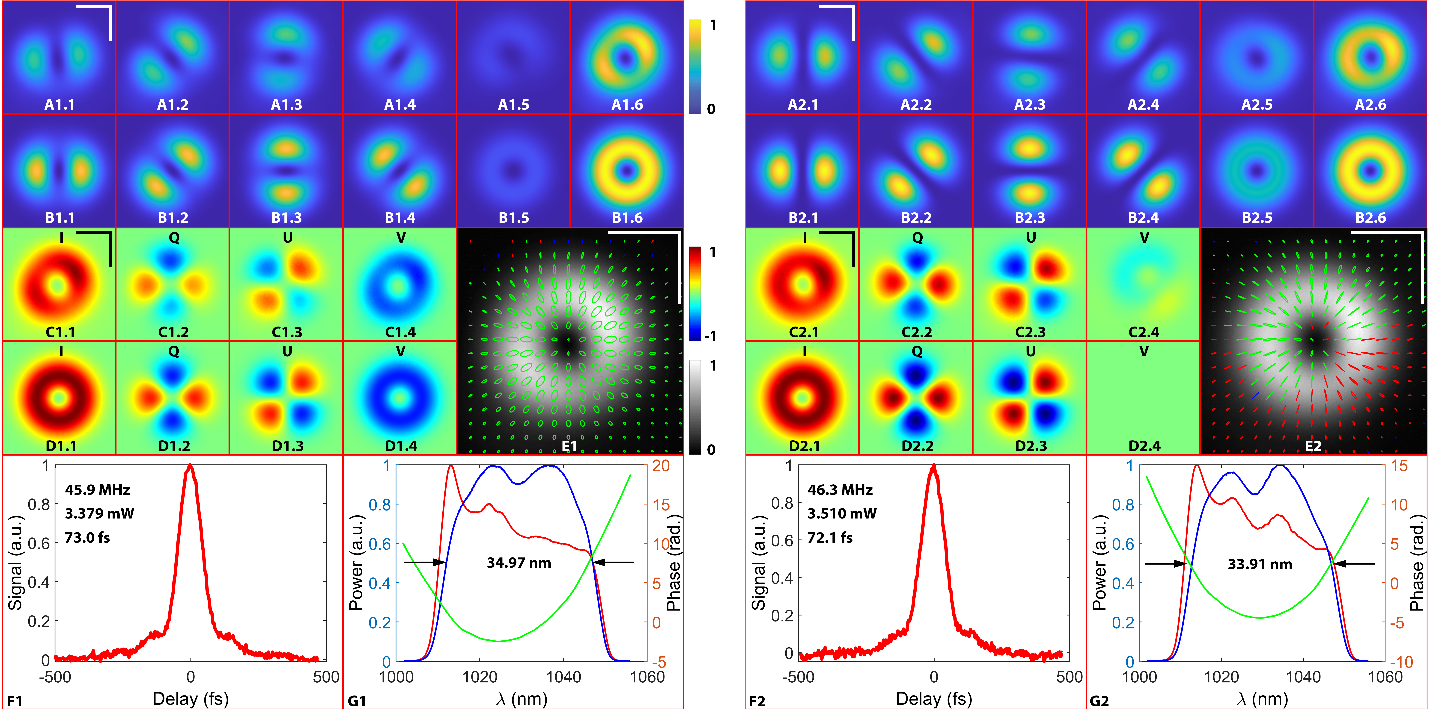


**I.3.E. Path E, the Circle of −45° Latitude**

This part demonstrates the characterizations of the states modulated along Path E. Table S2E shows the *T* and *E* values, and Figures 3E.1-3 are the conventional characterizations.

| **Index** | $\boldsymbol{\Phi}_{\mathbf{T}}$  **(rad.)** | $\boldsymbol{\Theta}_{\mathbf{T}}$  **(rad.)** | $\boldsymbol{\alpha}_{\mathbf{T}}$  **(rad.)** | $\boldsymbol{\beta}_{\mathbf{T}}$  **(rad.)** | $\boldsymbol{\alpha}_{\mathbf{T}}$  **(deg.)** | $\boldsymbol{\beta}_{\mathbf{T}}$  **(deg.)** | **Power (mW)** | $\boldsymbol{c}_{\boldsymbol{1}}$ | $\boldsymbol{c}_{\boldsymbol{2}}$ |
| --- | --- | --- | --- | --- | --- | --- | --- | --- | --- |
| **1** | 0 | $-\frac{\pi}{4}$ | $-\frac{\pi}{8}$ | $-\frac{\pi}{8}$ | 329.5 | 17.6 | 3.319 | $sin(\frac{\pi}{8})$ | $cos(\frac{\pi}{8})$ |
| **2** | $\frac{\pi}{4}$ | $-\frac{\pi}{4}$ | 0 | $-\frac{\pi}{8}$ | 352 | 17.6 | 3.194 | $sin(\frac{\pi}{8})exp(-\frac{\pi}{8}i)$ | $cos(\frac{\pi}{8})exp(\frac{\pi}{8}i)$ |
| **3** | $\frac{\pi}{2}$ | $-\frac{\pi}{4}$ | $\frac{\pi}{8}$ | $-\frac{\pi}{8}$ | 14.5 | 17.6 | 3.22 | $sin(\frac{\pi}{8})exp(-\frac{\pi}{4}i)$ | $cos(\frac{\pi}{8})exp(\frac{\pi}{4}i)$ |
| **4** | $\frac{3\pi}{4}$ | $-\frac{\pi}{4}$ | $\frac{\pi}{4}$ | $-\frac{\pi}{8}$ | 37 | 17.6 | 3.338 | $sin(\frac{\pi}{8})exp(-\frac{3\pi}{8}i)$ | $cos(\frac{\pi}{8})exp(\frac{3\pi}{8}i)$ |
| **5** | $\pi$ | $-\frac{\pi}{4}$ | $\frac{3\pi}{8}$ | $-\frac{\pi}{8}$ | 59.5 | 17.6 | 3.492 | $-sin(\frac{\pi}{8})i$ | $cos(\frac{\pi}{8})i$ |
| **6** | $\frac{5\pi}{4}$ | $-\frac{\pi}{4}$ | $\frac{\pi}{2}$ | $-\frac{\pi}{8}$ | 82 | 17.6 | 3.592 | $sin(\frac{\pi}{8})exp(-\frac{5\pi}{8}i)$ | $cos(\frac{\pi}{8})exp(\frac{5\pi}{8}i)$ |
| **7** | $\frac{3\pi}{2}$ | $-\frac{\pi}{4}$ | $\frac{5\pi}{8}$ | $-\frac{\pi}{8}$ | 104.5 | 17.6 | 3.542 | $sin(\frac{\pi}{8})exp(-\frac{3\pi}{4}i)$ | $cos(\frac{\pi}{8})exp(\frac{3\pi}{4}i)$ |
| **8** | $\frac{7\pi}{4}$ | $-\frac{\pi}{4}$ | $\frac{3\pi}{4}$ | $-\frac{\pi}{8}$ | 127 | 17.6 | 3.446 | $sin(\frac{\pi}{8})exp(-\frac{7\pi}{8}i)$ | $cos(\frac{\pi}{8})exp(\frac{7\pi}{8}i)$ |
| **9** | $2\pi$ | $-\frac{\pi}{4}$ | $\frac{7\pi}{8}$ | $-\frac{\pi}{8}$ | 149.5 | 17.6 | 3.379 | $-sin(\frac{\pi}{8})$ | $-cos(\frac{\pi}{8})$ |
| **10** | 2$\pi$ | 0 | 0 | 0 | 352 | 355.1 | 3.51 | $-\frac{1}{\sqrt{2}}$ | $-\frac{1}{\sqrt{2}}$ |

**Table S2E.** List of *T* and *E* values for $\left| -1,L \right\rangle$ and $\left| +1,R \right\rangle$ modulated along Path E.
