# Supplementary material for: Integrated pulse scope for tunable generation and intrinsic characterization of structured femtosecond laser
Source: Sci Rep. 2021 May 6;11:9670. doi: 10.1038/s41598-021-87938-w (PMC8102529; doi:10.1038/s41598-021-87938-w)
Supplement: Supplementary file 4 — Supplementary Information 4. [file 41598_2021_87938_MOESM4_ESM.docx]

**Figure S3E1.** Conventional characterizations of the states $\left( 0\boldsymbol{,-}\pi/4 \right)$, $\left( \pi/4,-\pi/4 \right)$, $\left( \pi/2,-\pi/4 \right)$, and $\left( 3\pi/4,-\pi/4 \right)$ on $\overline{S}$. **A1-G1**: [$sin(\frac{\pi}{8})\left| -1,L \right\rangle+cos(\frac{\pi}{8})\left| +1,R \right\rangle]/\sqrt{2}$ corresponding to the radial state $\left( 0\boldsymbol{,-}\pi/4 \right)$. **A2-G2**: $[sin(\frac{\pi}{8})exp(-\frac{\pi}{8}i)\left| -1,L \right\rangle+cos(\frac{\pi}{8})exp(\frac{\pi}{8}i)\left| +1,R \right\rangle]/\sqrt{2}$ corresponding to the state $\left( \pi/4,-\pi/4 \right)$. **A3-G3**: $[sin(\frac{\pi}{8})exp(-\frac{\pi}{4}i)\left| -1,L \right\rangle+cos(\frac{\pi}{8})exp(\frac{\pi}{4}i)\left| +1,R \right\rangle]/\sqrt{2}$ corresponding to the point $\left( \pi/2,-\pi/4 \right)$. **A4-G4**:$[sin(\frac{\pi}{8})exp(-\frac{3\pi}{8}i)\left| -1,L \right\rangle+cos(\frac{\pi}{8})exp(\frac{3\pi}{8}i)\left| +1,R \right\rangle]/\sqrt{2}$ corresponding to the point $\left( 3\pi/4,-\pi/4 \right)$.


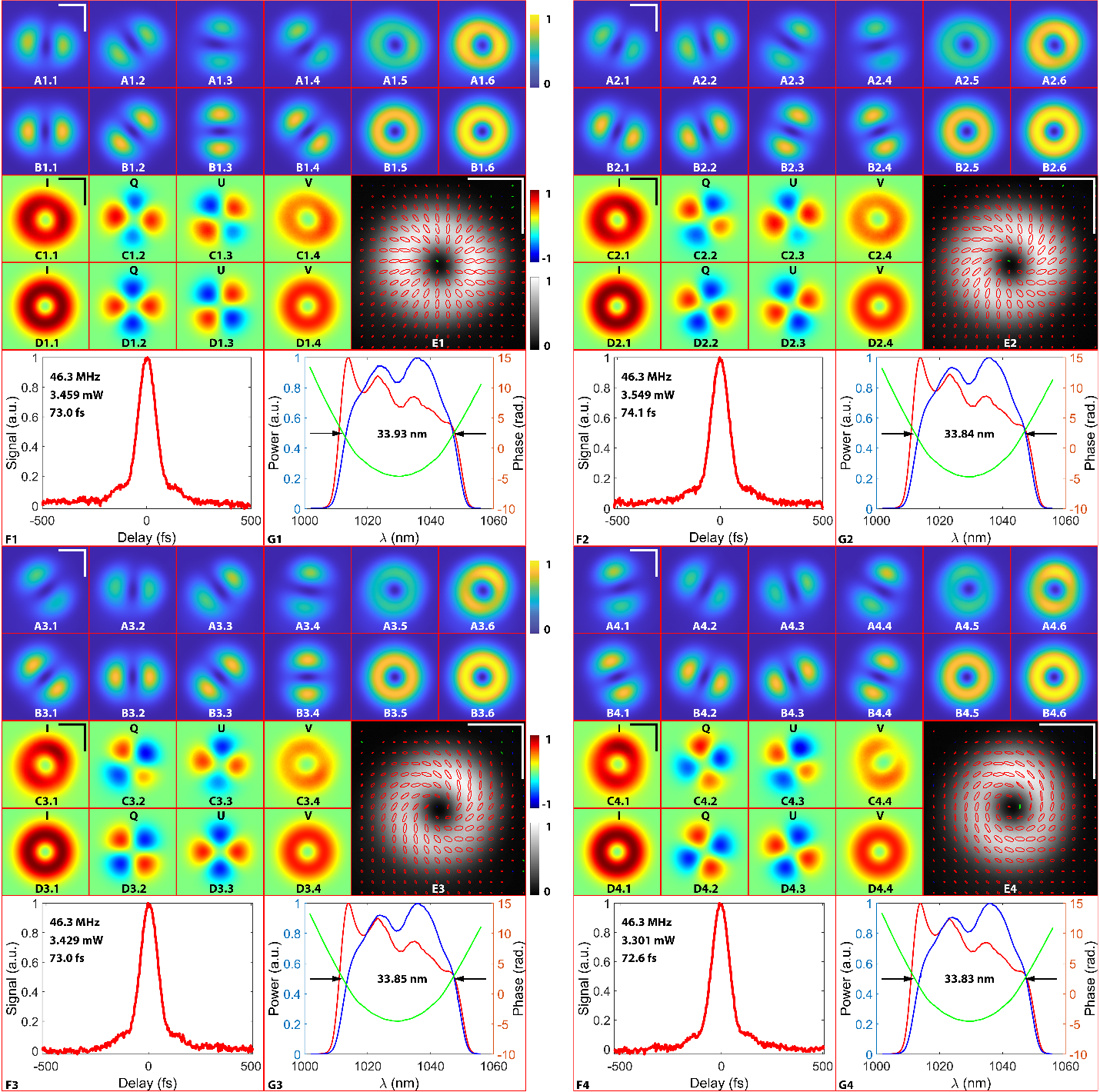


**Figure S3E2.** Conventional characterizations of the states described by $\left( \pi\boldsymbol{,-}\pi/4 \right)$, $\left( \frac{5\pi}{4},-\pi/4 \right)$, $\left( \frac{3\pi}{2},-\pi/4 \right)$, and $\left( \frac{7\pi}{4},-\pi/4 \right)$ on $\overline{S}$. **A1-G1**: $[-sin\left( \frac{\pi}{8} \right)\left| -1,L \right\rangle+cos\left( \frac{\pi}{8} \right)\left| +1,R \right\rangle]i/\sqrt{2}$ corresponding to the radial state $\left( \pi\boldsymbol{,-}\frac{\pi}{4} \right)$. **A2-G2**: $[sin(\frac{\pi}{8})exp(-\frac{5\pi}{8}i)\left| -1,L \right\rangle+cos(\frac{\pi}{8})exp(\frac{5\pi}{8}i)\left| +1,R \right\rangle]/\sqrt{2}$ corresponding to the state $\left( \frac{5\pi}{4},-\pi/4 \right)$. **A3-G3**: $[sin(\frac{\pi}{8})exp(-\frac{3\pi}{4}i)\left| -1,L \right\rangle+cos(\frac{\pi}{8})exp(\frac{3\pi}{4}i)\left| +1,R \right\rangle]/\sqrt{2}$ corresponding to the point $\left( \frac{3\pi}{2},-\pi/4 \right)$. **A4-G4**:$[sin(\frac{\pi}{8})exp(-\frac{7\pi}{8}i)\left| -1,L \right\rangle+cos(\frac{\pi}{8})exp(\frac{7\pi}{8}i)\left| +1,R \right\rangle]/\sqrt{2}$ corresponding to the point $\left( \frac{7\pi}{4},-\pi/4 \right)$.


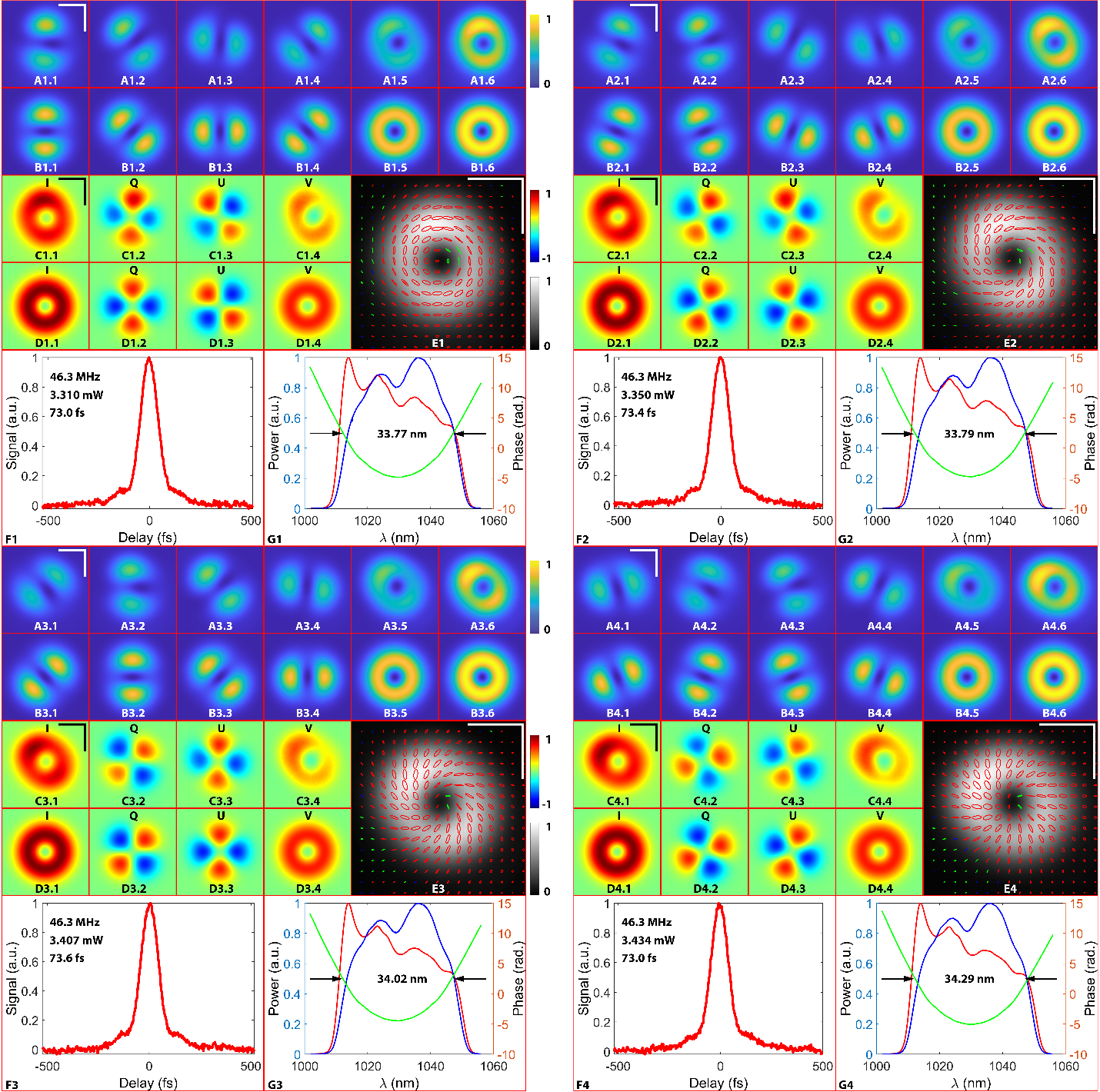

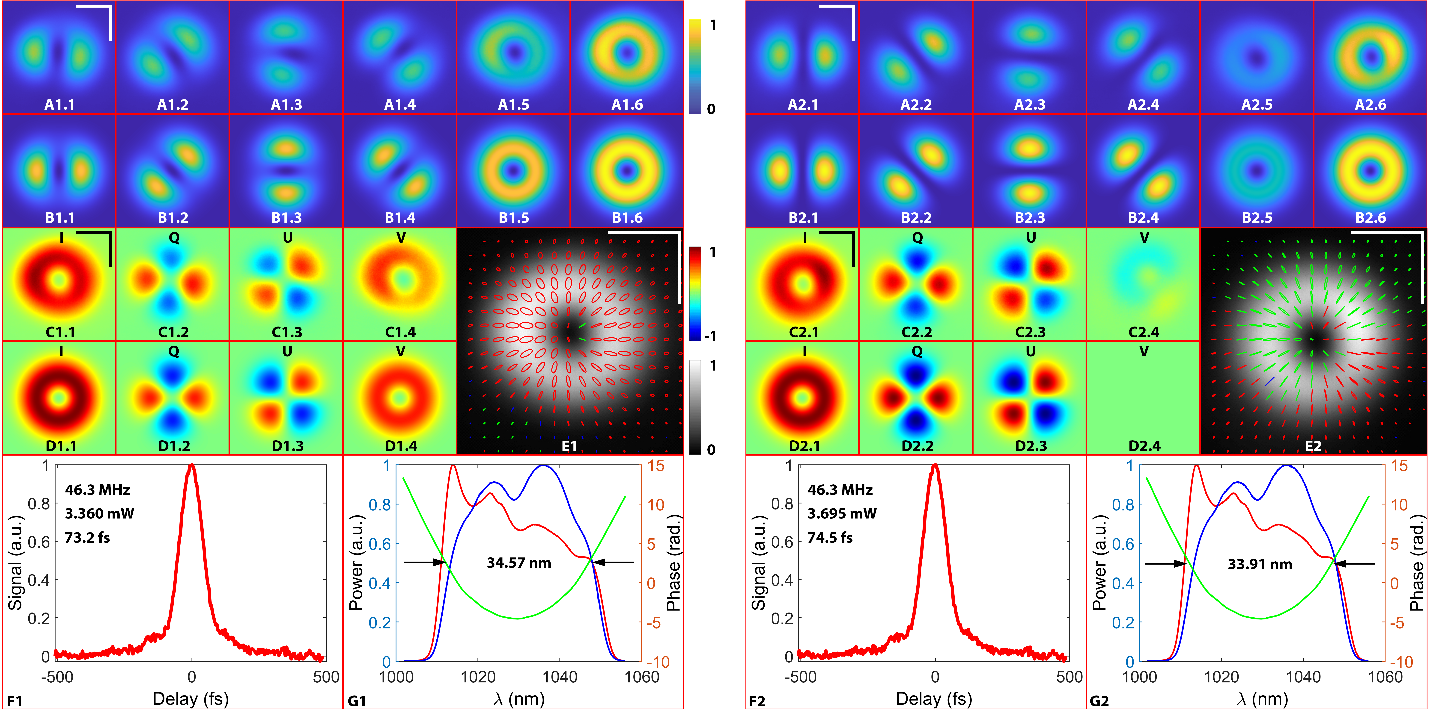


**Figure S3E3.** Conventional characterizations of the states $\left( 2\pi\boldsymbol{,-}\pi/4 \right)$ and $\left( 2\pi,0 \right)$ on $\overline{S}$. **A1-G1**: $-[sin\left( \frac{\pi}{8} \right)\left| -1,L \right\rangle+cos\left( \frac{\pi}{8} \right)\left| +1,R \right\rangle]/\sqrt{2}$ corresponding to the radial state $\left( 2\pi\boldsymbol{,}\pi/4 \right)$. **A2-G2**: $-(\left| -1,L \right\rangle+\left| +1,R \right\rangle)/\sqrt{2}$ corresponding to the state $\left( 2\pi,0 \right)$.


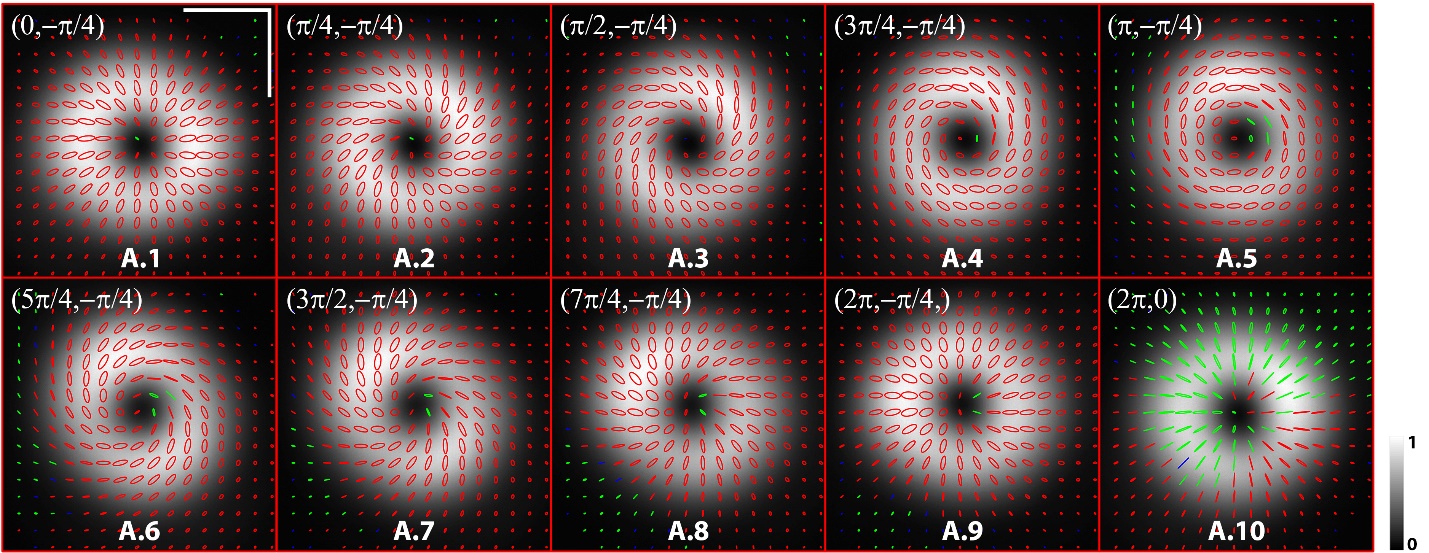


**Figure S3G.** The polarization ellipses of a pulse continually modulated along Path E on the HOP sphere. Green denotes the left-handed polarization, red the right-handed polarization, and blue the linearly polarized. The radial point state was used as the starting point, hence the 10 measurement points in Path E. Scale bar represents 1 mm.


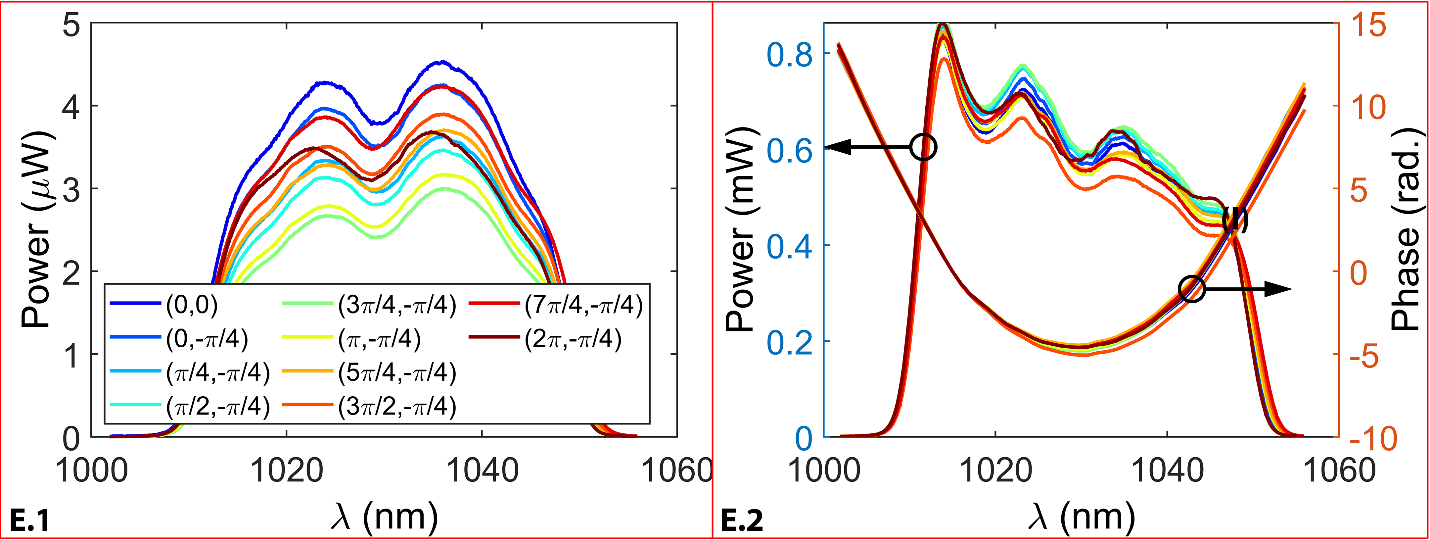


**Figure S3F.** Spectrum and phase measurements of the pulse continually modulated along Path E on the HOP sphere. Every spherical coordinate ($\Phi,\Theta$) in the legend denotes a measurement point. Roman numeral I denotes Zone I.

**Section II: Detailed numerical processing for obtaining the spatiospectral and spatiotemporal properties of the femtosecond pulses**

**I.1. The principles of the spatiotemporal pulse characterization**

On plane $\boldsymbol{\Pi}$, the time domain and spectral domain electric field of the reference beam can be denoted by a three components matrix:

|  | $\boldsymbol{E}^{ref}(\boldsymbol{r},t)=\left( \begin{matrix} E_{x}^{ref}(\boldsymbol{r},t) \\ E_{y}^{ref}(\boldsymbol{r},t) \\ 0 \end{matrix} \right)$, and ${\tilde{\boldsymbol{E}}}^{ref}\left( \boldsymbol{r},\omega\right)=\left( \begin{matrix} \tilde{E}_{x}^{ref}(\boldsymbol{r},\omega) \\ \tilde{E}_{y}^{ref}(\boldsymbol{r},\omega) \\ 0 \end{matrix} \right)$ | **(S7)** |
| --- | --- | --- |

and for the sample beam:

|  | $\boldsymbol{E}^{sam}(\boldsymbol{r},t)=\left( \begin{matrix} E_{x}^{sam}(\boldsymbol{r},t) \\ E_{y}^{sam}(\boldsymbol{r},t) \\ E_{z}^{sam}(\boldsymbol{r},t) \end{matrix} \right)$, and ${\tilde{\boldsymbol{E}}}^{sam}\left( \boldsymbol{r},\omega\right)=\left( \begin{matrix} \tilde{E}_{x}^{sam}(\boldsymbol{r},\omega) \\ \tilde{E}_{y}^{sam}(\boldsymbol{r},\omega) \\ \tilde{E}_{z}^{sam}(\boldsymbol{r},\omega) \end{matrix} \right)$ | **(S8)** |
| --- | --- | --- |

Based on the semiclassical theory of photoelectric detection of light, the averaged intensity at point $\boldsymbol{r}$ on $\boldsymbol{\Pi}$ becomes, under the assumption of stationarity and ergodicity:

$$I\left( \boldsymbol{r},\tau\right)=\left\langle{(\boldsymbol{E}^{sam}\left( \boldsymbol{r},t \right)+\boldsymbol{E}^{ref}\left( \boldsymbol{r},t+\tau\right))}^{*}\cdot(\boldsymbol{E}^{sam}\left( \boldsymbol{r},t \right)+\boldsymbol{E}^{ref}\left( \boldsymbol{r},t+\tau\right)) \right\rangle=\lim_{T\to\infty} \frac{1}{2T}\int_{-T}^{T} \left( \boldsymbol{E}^{sam}\left( \boldsymbol{r},t \right)+\boldsymbol{E}^{ref}\left( \boldsymbol{r},t+\tau\right) \right)^{*}\cdot\left( \boldsymbol{E}^{sam}\left( \boldsymbol{r},t \right)+\boldsymbol{E}^{ref}\left( \boldsymbol{r},t+\tau\right) \right)dt$$

|  | $=\left\langle{\vert\boldsymbol{E}^{sam}\left( \boldsymbol{r},t \right)\vert}^{2} \right\rangle+\left\langle{\vert\boldsymbol{E}^{ref}\left( \boldsymbol{r},t \right)\vert}^{2} \right\rangle+2Re(\left\langle{(\boldsymbol{E}^{sam}\left( \boldsymbol{r},t \right))}^{*}\cdot(\boldsymbol{E}^{ref}\left( \boldsymbol{r},t+\tau\right)) \right\rangle)$ | **(S9)** |
| --- | --- | --- |

By applying the Fourier transformation ($\mathcal{F}$), Eq. S6 can be separated into 3 terms:

$$\tilde{I}\left( \boldsymbol{r},\omega\right)\mathcal{=F}\left[ \left\langle\left| \boldsymbol{E}^{sam}\left( \boldsymbol{r},t \right) \right|^{2} \right\rangle+\left\langle\left| \boldsymbol{E}^{ref}\left( \boldsymbol{r},t \right) \right|^{2} \right\rangle\right]+\left( {\tilde{\boldsymbol{E}}}^{sam}\left( \boldsymbol{r},\omega\right) \right)^{*}\cdot\left( {\tilde{\boldsymbol{E}}}^{ref}\left( \boldsymbol{r},\omega\right) \right)+{({\tilde{\boldsymbol{E}}}^{ref}\left( \boldsymbol{r},-\omega\right))}^{*}\cdot({\tilde{\boldsymbol{E}}}^{sam}\left( \boldsymbol{r},-\omega\right))$$

i.e., the DC term and two mirror terms which can be separated into the spectral domain by using the super-gaussian spectral filter, then the second component can be obtained as:

|  | $\tilde{S}\left( \boldsymbol{r},\omega\right)={({\tilde{\boldsymbol{E}}}^{ref}\left( \boldsymbol{r},\omega\right))}^{*}\cdot({\tilde{\boldsymbol{E}}}^{sam}\left( \boldsymbol{r},\omega\right))$ | **(S10)** |
| --- | --- | --- |

Therefore, by rotating the polarizer, the horizontal and vertical channels of the signals can be detected by the CCD as:

|  | $\left\{ \begin{aligned} \tilde{S}_{x}\left( \boldsymbol{r},\omega\right)={(\tilde{E}_{x}^{ref}(\boldsymbol{r},\omega))}^{*}(\tilde{E}_{x}^{sam}(\boldsymbol{r},\omega))e^{i\varphi_{x0}} \\ \tilde{S}_{y}\left( \boldsymbol{r},\omega\right)={(\tilde{E}_{y}^{ref}(\boldsymbol{r},\omega))}^{*}(\tilde{E}_{y}^{sam}(\boldsymbol{r},\omega))e^{i\varphi_{y0}} \end{aligned} \right.$ | **(S11)** |
| --- | --- | --- |

where $\varphi_{x0}$ and $\varphi_{y0}$ are the random phase corresponding to two independent polarization channels. This equation can be rewritten as:

|  | $\left\{ \begin{aligned} \tilde{E}_{x}^{sam}(\boldsymbol{r},\omega)=\tilde{S}_{x}\left( \boldsymbol{r},\omega\right)e^{-i\varphi_{x0}}/{(\tilde{E}_{x}^{ref}(\boldsymbol{r},\omega))}^{*} \\ \tilde{E}_{y}^{sam}(\boldsymbol{r},\omega)=\tilde{S}_{y}\left( \boldsymbol{r},\omega\right)e^{-i\varphi_{y0}}/{(\tilde{E}_{y}^{ref}(\boldsymbol{r},\omega))}^{*} \end{aligned} \right.$ | **(S12)** |
| --- | --- | --- |

The time domain electric field of the sample can be given by:

|  | $\left\{ \begin{aligned} E_{x}^{sam}\left( \boldsymbol{r},t \right)\mathcal{=inF}{[\tilde{S}}_{x}\left( \boldsymbol{r},\omega\right)e^{-i\varphi_{x0}}/\left( \tilde{E}_{x}^{ref}\left( \boldsymbol{r},\omega\right) \right)^{*}] \\ E_{y}^{sam}\left( \boldsymbol{r},t \right)\mathcal{=inF}{[\tilde{S}}_{y}\left( \boldsymbol{r},\omega\right)e^{-i\varphi_{y0}}/\left( \tilde{E}_{y}^{ref}\left( \boldsymbol{r},\omega\right) \right)^{*}] \end{aligned} \right.$ | **(S13)** |
| --- | --- | --- |

where symbol “$\mathcal{inF}$” denotes the inverse Fourier transformation. Therefore, providing a homogenous reference beam across the whole detection plane of the CCD, the knowledges the spectrum and phase by using the optical spectrum analyzer and FROG (laboratory-built, based on a BBO crystal), the whole spectral domain information of the sample pulse can be obtained by using the Eq. S12. The temporal structure of the pulse can be rebuild based on the Eq. S13 after the inverse Fourier transformation.
