# Supplementary material for: Integrated pulse scope for tunable generation and intrinsic characterization of structured femtosecond laser
Source: Sci Rep. 2021 May 6;11:9670. doi: 10.1038/s41598-021-87938-w (PMC8102529; doi:10.1038/s41598-021-87938-w)
Supplement: Supplementary file 5 — Supplementary Information 5. [file 41598_2021_87938_MOESM5_ESM.docx]

**I.2. The experimental 2D interferograms recorded using the polarization-sensitive time-scanning MZI at a single time delay**

For the space–time pulses characterizations, Figure S4 demonstrates the 2D interferograms recorded with the polarization-sensitive time-scanning MZI for different HOP_SS_ pulses. In Figure S4A, the red circles denote the well-defined singularities of the north and south pole states. It be found that the north pole state (0,π/2) has the helicity of −1, and the south pole state (0, −π/2) has the helicity of +1. Furthermore, the state on the Equator, the radial, azimuthal, the state for the (π/2,0) and (3π/2,0) (shown in Figure S4B and Figure S4C) keep a homogeneous interference patterns which coincide with the principle of the Q-plate. The inhomogeneous intensity distribution on the 2D interferograms are produced by the special shape of the donut-like pulse as well as the sectioning procedure of the time scanning techniques, whose details can be found in the 3D interferograms of Figure 5.

**Figure S4A.** The experimental 2D interferograms recorded with the polarization sensitive time scanning MZI for different HOP_SS_ pulses. **A**: 2D interferograms for north pole state at time delay between the two interferometer’s arms of 546.38 fs. **A.1** and **A.2** corresponding to the *H* and *V* channels, respectively. **A.3** is single-pixel cross-correlation signal obtained from the time scanning procedure which containing 1700 steps. **B**: 2D interferograms for south pole state at time delay of 546.38 fs. The red circle denotes the well-defined singularities of the HOP_SS_ pulses. Scale bars represent 1 mm.


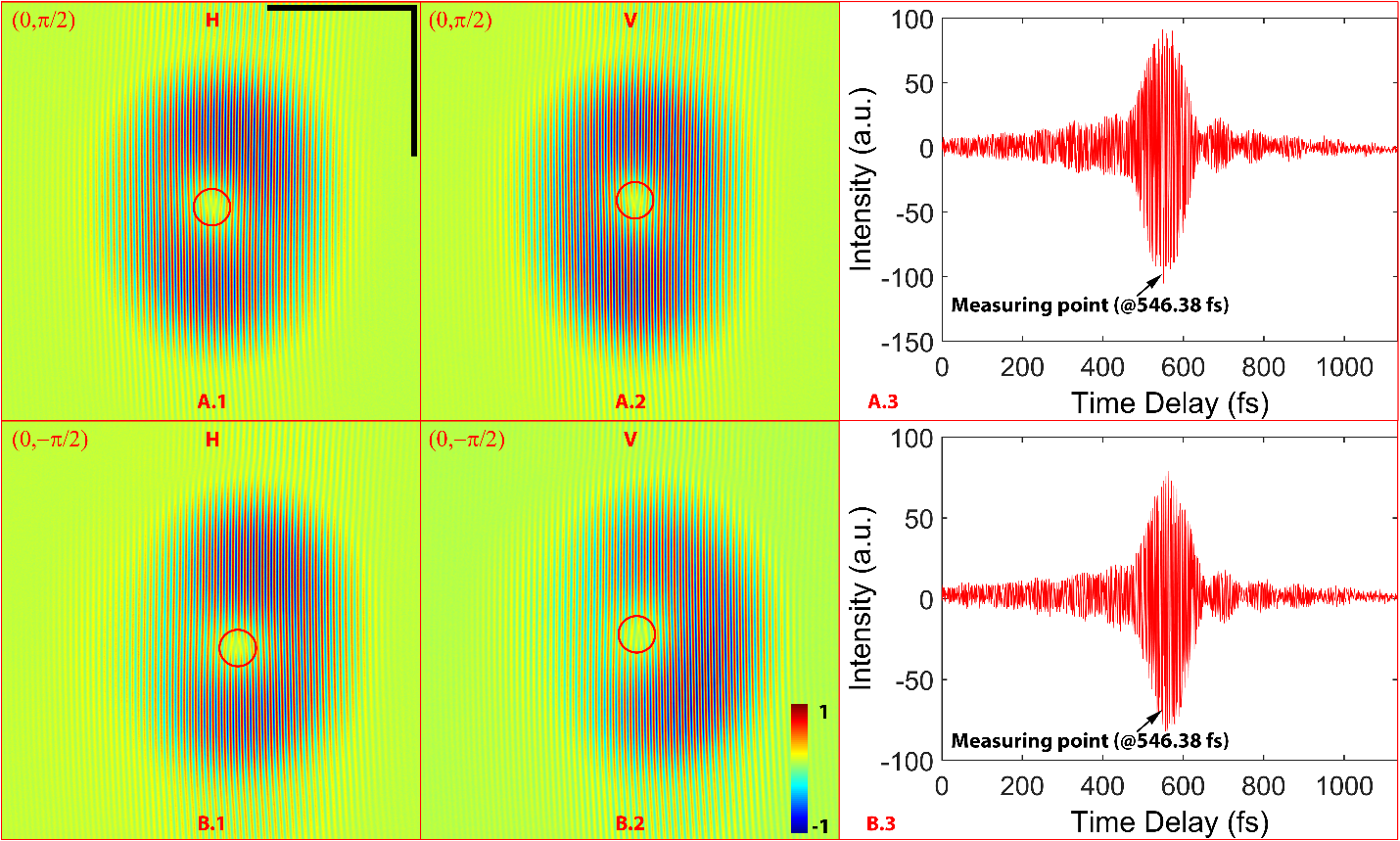


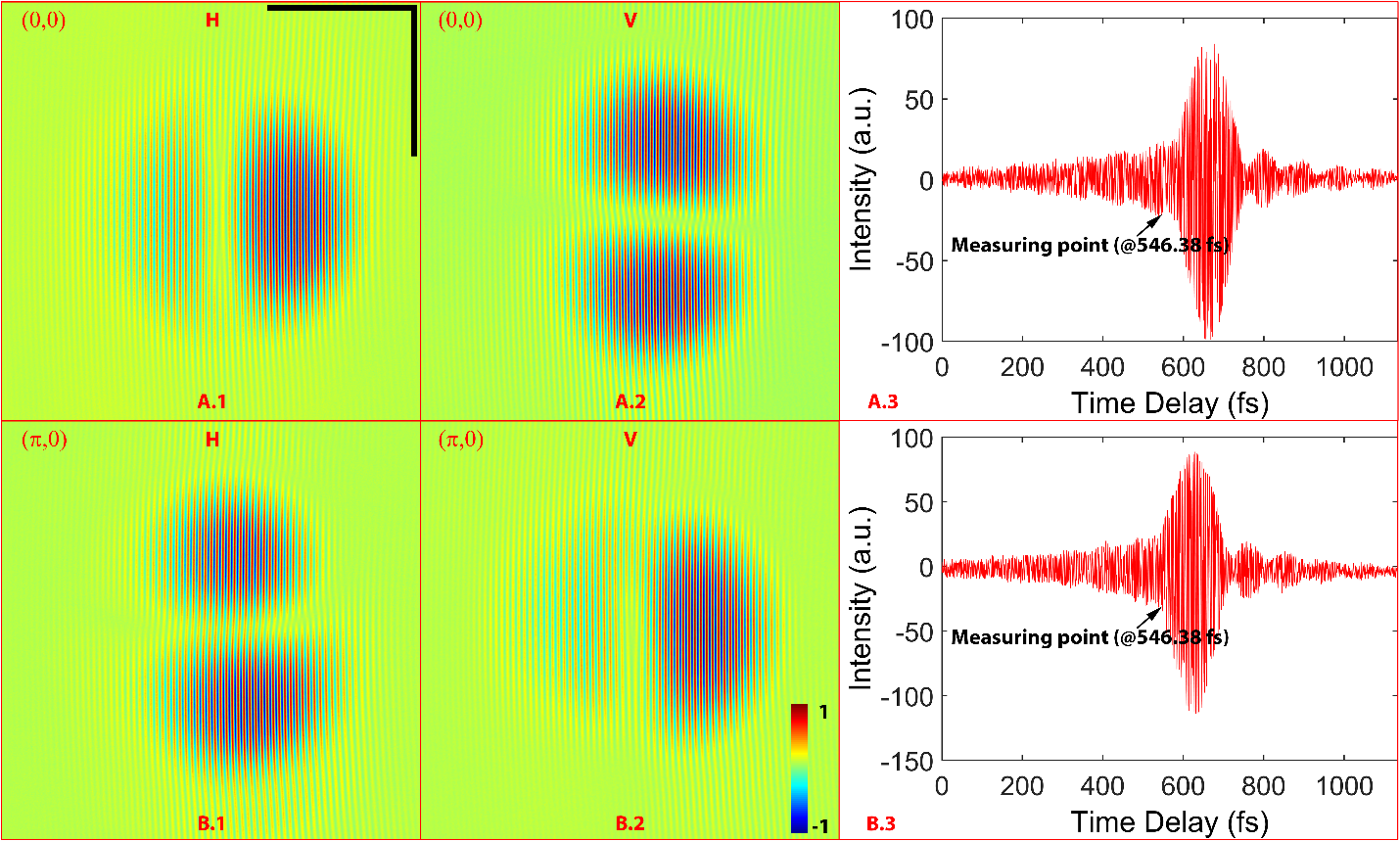


**Figure S4B.** The experimental 2D interferogram results. **A**: 2D interferograms of the radial state at time delay between the two interferometer’s arms of 546.38 fs. **A.1** and **A.2** corresponding to the *H* and *V* channels, respectively. **A.3** is single-pixel cross-correlation signal obtained from the time scanning procedure. **B**: 2D interferograms of the azimuthal state at time delay of 546.38 fs. Scale bars represent 1 mm.


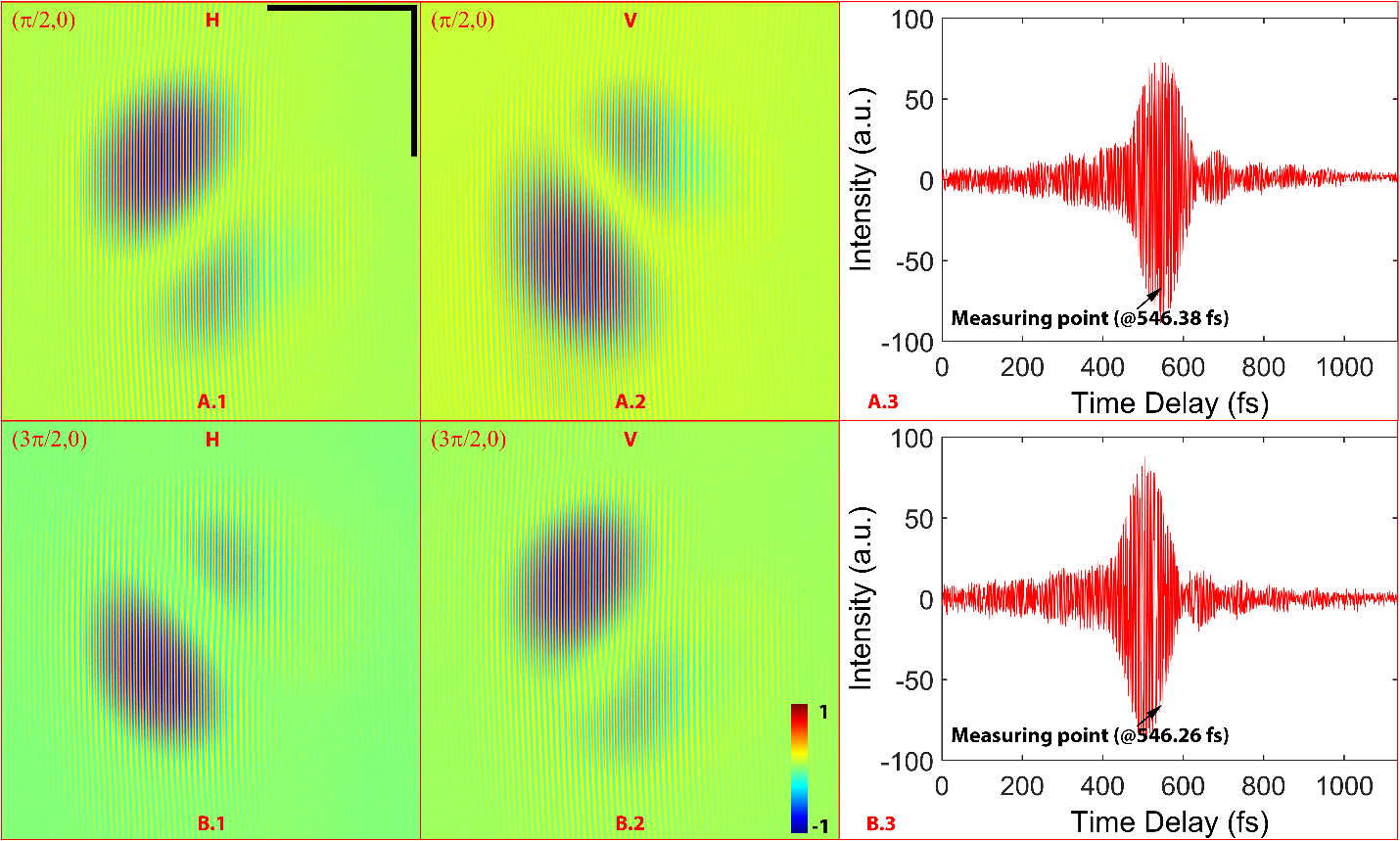


**Figure S4C.** The experimental 2D interferogram results. **A**: 2D interferograms for $(\pi/2,0)$ state at time delay between the two interferometer’s arms of 546.38 fs. **A.1** and **A.2** corresponding to the *H* and *V* channels, respectively. **A.3** is single-pixel cross-correlation signal obtained from the time scanning procedure. **B**: 2D interferograms for $(3\pi/2,0)$ state at time delay of 546.26 fs. Scale bars represent 1 mm.

**I.3. The additional spectral characterization of the femtosecond pulse with HOP_SS_**

Figures S5A-E demonstrate additional polarization-sensitive spatial-temporal characterization of the femtosecond pulse with the states of south pole (0,-π/2), radial (0,0), azimuthal (π,0), (π/2,0) and (3π/2,0) of the HOP sphere.


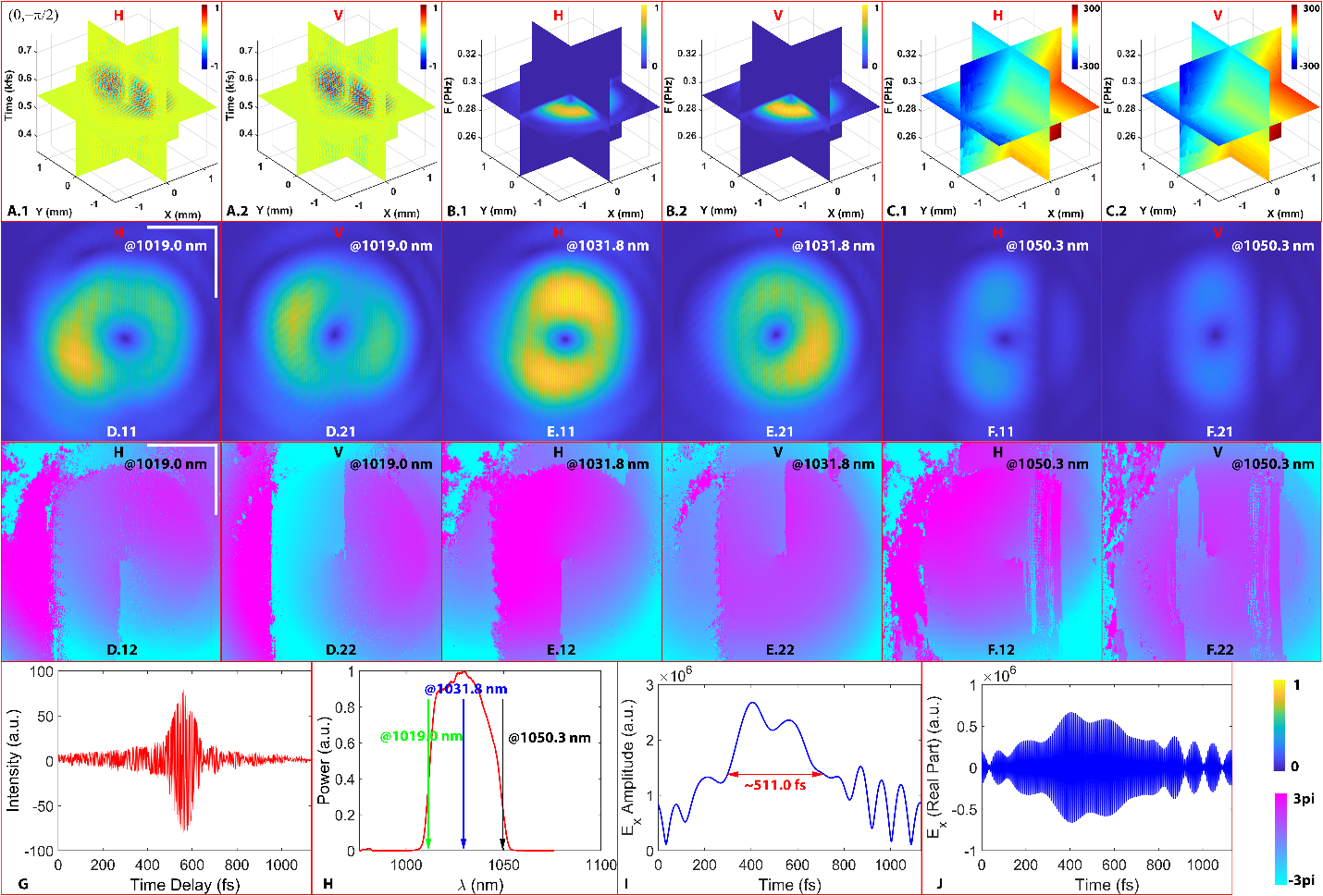


**Figure S5A.** The polarization-sensitive spatiotemporal characterizations of the pulse with south pole state $(0, -\pi/2)$. **A1-2**: the 3D interferograms of the horizontal *H* and *V* channels, respectively; **B1-2**: the corresponding 3D spectral amplitude of the two channels; **C1-2**: the corresponding 3D spectral phase. **D, E,** and **F**: the 2D amplitude (Roman numeral i) and unwrapped phase profiles (Roman numeral ii) at 1019.0 nm, 1031.8 nm and 1050.3 nm, respectively. **G**: single-pixel cross-correlation signal obtained from the time scanning procedure for *H* channel; **H**: the single-pixel spectrum; **I**: the single-pixel profile for the total temporal amplitude envelope; **J**: the corresponding real part of the pulse. Scale bars represent 1 mm.


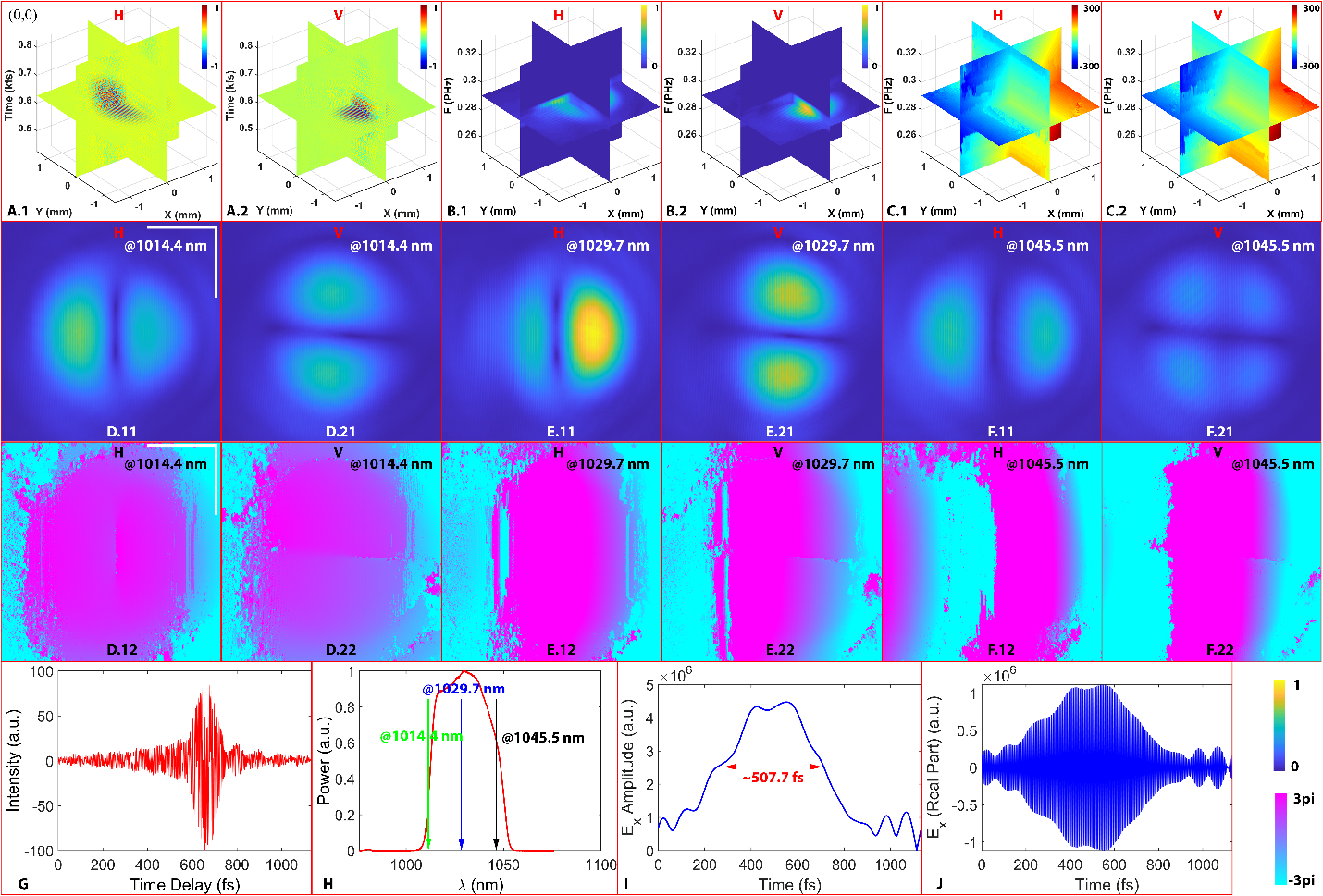


**Figure S5B.** The polarization-sensitive spatiotemporal characterization of the pulse with radial state $(0,0)$. **A1-2**: the 3D interferograms of the *H* and vertical *V* channels, respectively; **B1-2**: the corresponding 3D spectral amplitude of the two channels; **C1-2**: the corresponding 3D spectral phase. **D, E,** and **F**: the 2D amplitude (Roman numeral i) and unwrapped phase profiles (Roman numeral ii) at 1014.4 nm, 1029.7 nm and 1045.5 nm, respectively. **G**: single-pixel cross-correlation signal obtained from the time scanning procedure for *H* channel; **H**: the single-pixel spectrum; **I**: the single-pixel profile for the total temporal amplitude envelope; **J**: the corresponding real part of the pulse. Scale bars represent 1 mm.


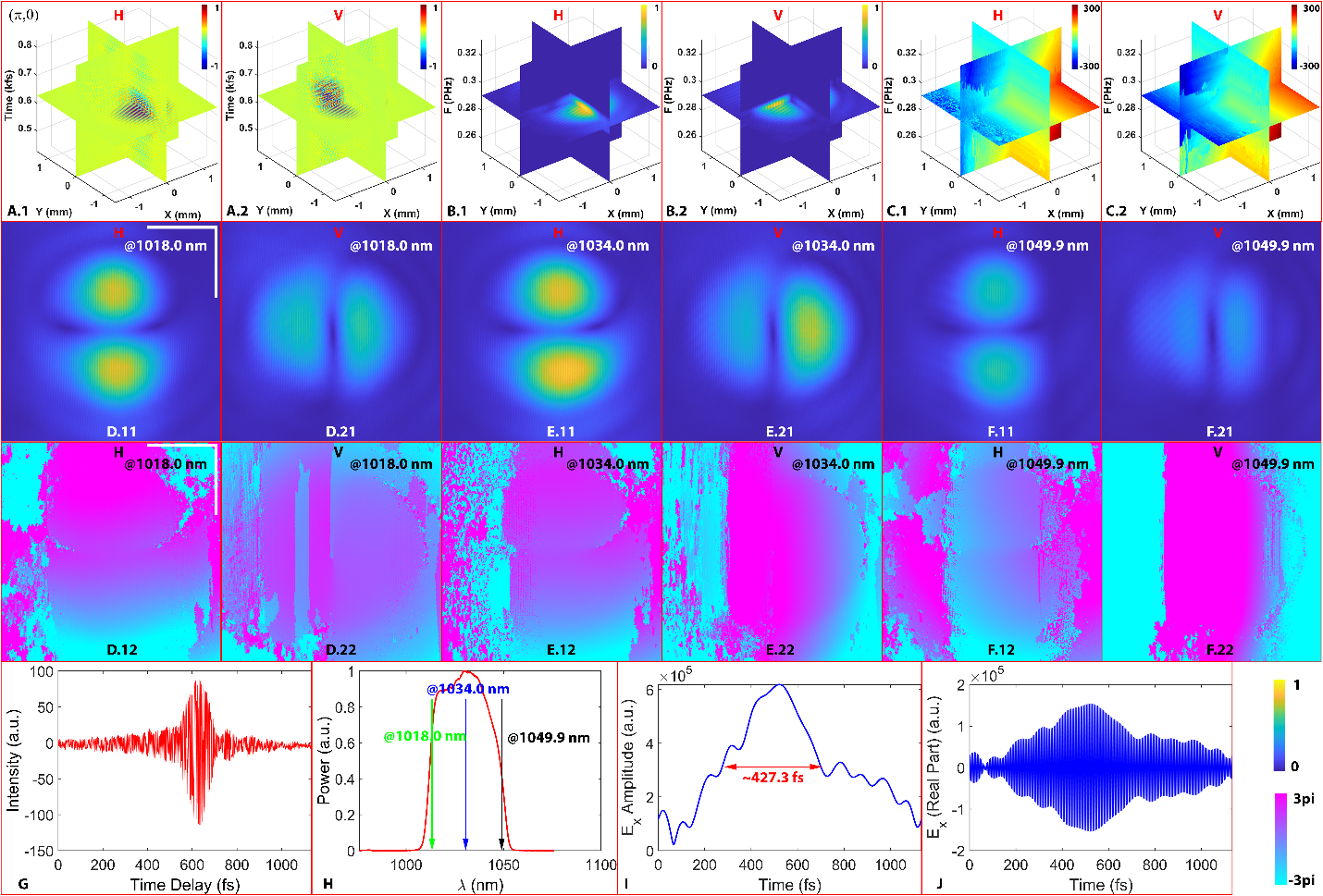


**Figure S5C.** The polarization-sensitive spatiotemporal characterizations of the pulse with azimuthal state $(\pi,0)$. **A1-2**: the 3D interferograms of the *H* and *V* channels, respectively; **B1-2**: the corresponding 3D spectral amplitude of the two channels; **C1-2**: the corresponding 3D spectral phase. **D, E,** and **F**: the 2D amplitude (Roman numeral i) and unwrapped phase profiles (Roman numeral ii) at 1018.0 nm, 1034.0 nm and 1049.9 nm, respectively. **G**: single-pixel cross-correlation signal obtained from the time scanning procedure for *H* channel; **H**: the single-pixel spectrum; **I**: the single-pixel profile for the total temporal amplitude envelope; **J**: the corresponding real part of the pulse. Scale bars represent 1 mm.


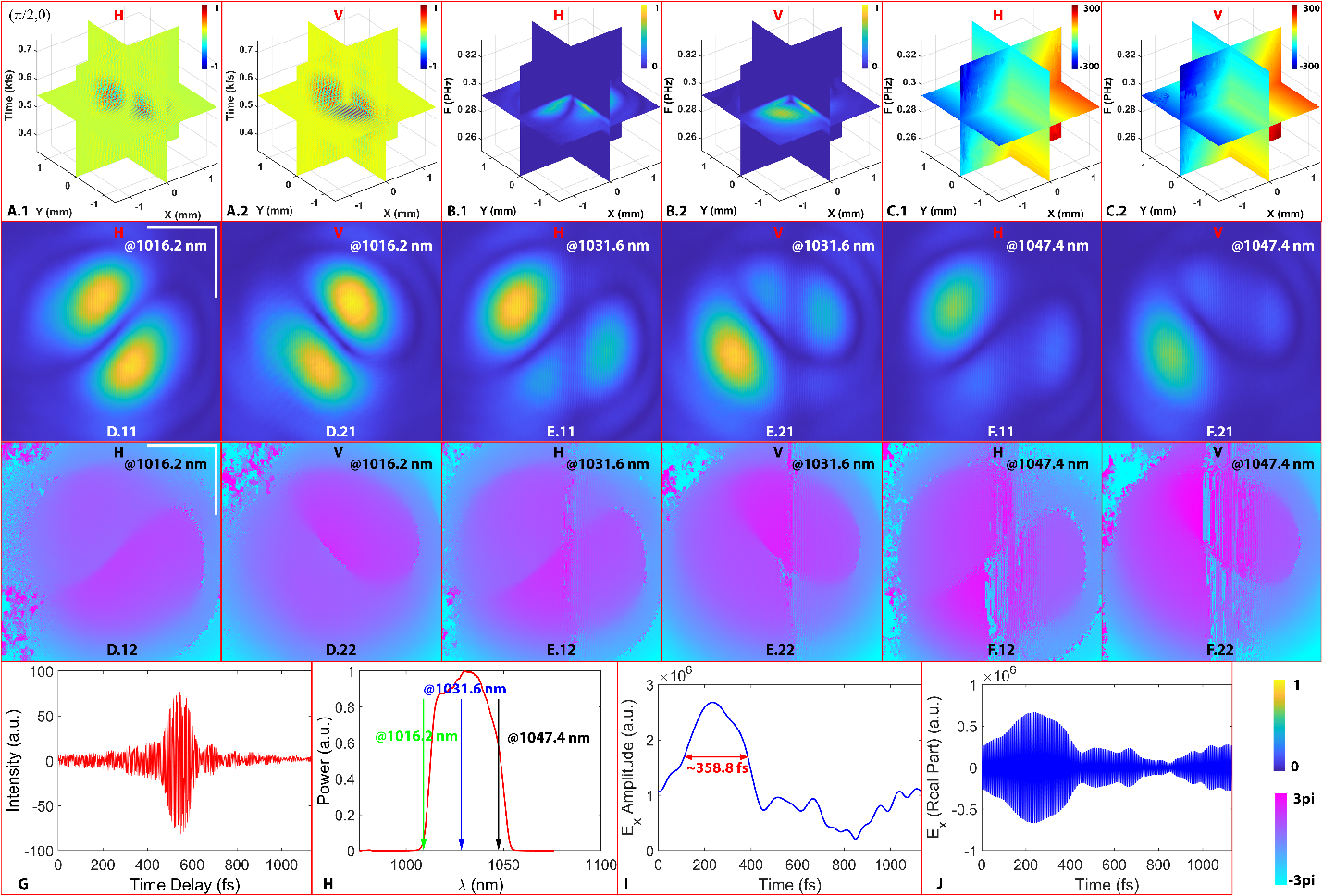


**Figure S5D.** The polarization-sensitive spatiotemporal characterizations of the pulse with $(\pi/2,0)$ state of the HOP sphere. **A1-2**: the 3D interferograms of the *H* and *V* channels, respectively; **B1-2**: the corresponding 3D spectral amplitude of the two channels; **C1-2**: the corresponding 3D spectral phase. **D, E,** and **F**: the 2D amplitude (Roman numeral i) and unwrapped phase profiles (Roman numeral ii) at 1016.2 nm, 1031.6 nm and 1047.4 nm, respectively. **G**: single-pixel cross-correlation signal obtained from the time scanning procedure for *H* channel; **H**: the single-pixel spectrum; **I**: the single-pixel profile for the total temporal amplitude envelope; **J**: the corresponding real part of the pulse. Scale bars represent 1 mm.


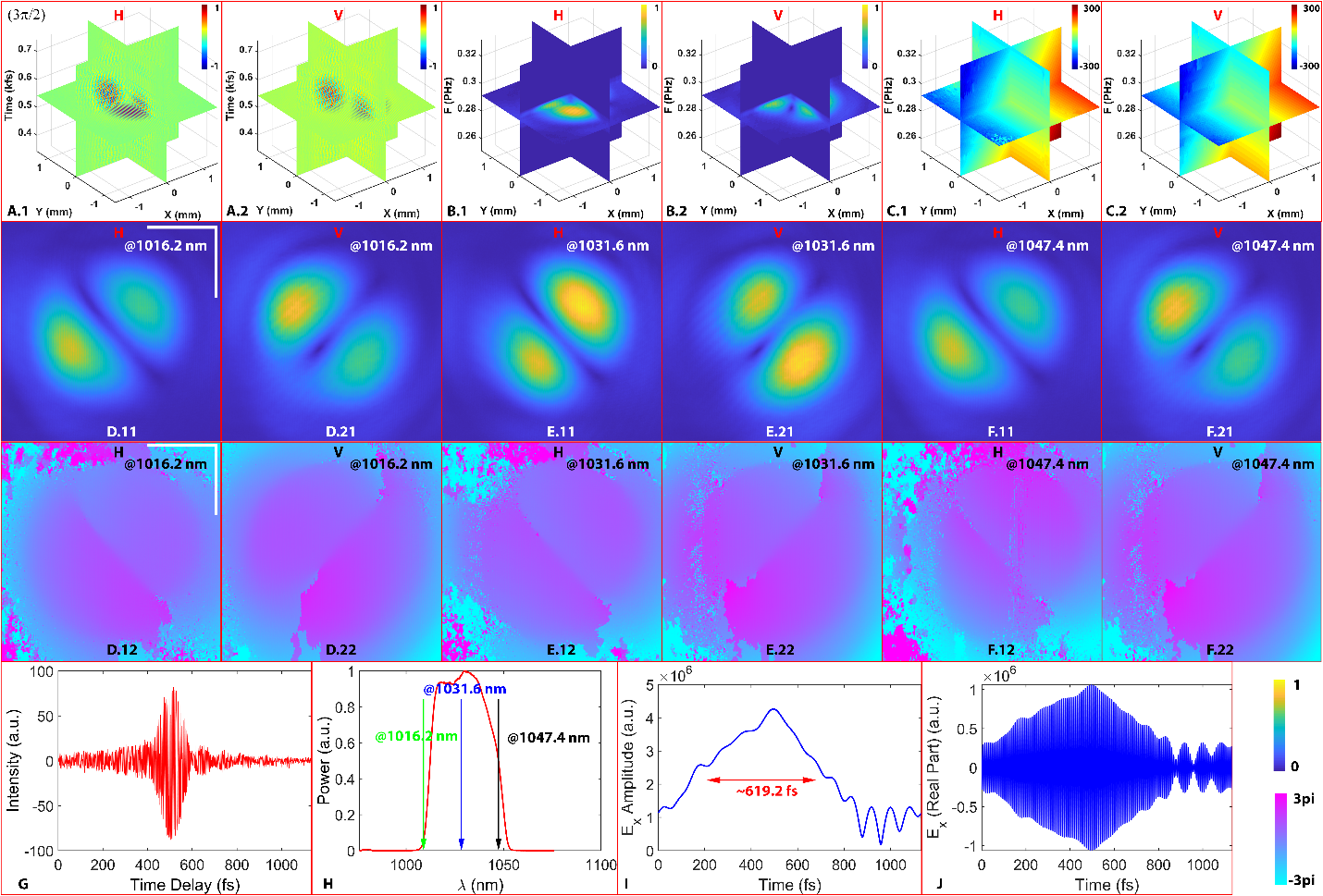


**Figure S5E.** The polarization-sensitive spatiotemporal characterizations of the pulse with $(3\pi/2,0)$ state of the HOP sphere. **A1-2**: the 3D interferograms of the *H* and *V* channels, respectively; **B1-2**: the corresponding 3D spectral amplitude of the two channels; **C1-2**: the corresponding 3D spectral phase. **D, E,** and **F**: the 2D amplitude (Roman numeral i) and unwrapped phase profiles (Roman numeral ii) at 1016.2 nm, 1031.6 nm and 1047.4 nm, respectively. **G**: single-pixel cross-correlation signal obtained from the time scanning procedure for *H* channel; **H**: the single-pixel spectrum; **I**: the single-pixel profile for the total temporal amplitude envelope; **J**: the corresponding real part of the pulse. Scale bars represent 1 mm.
